# Integrating Single‐Cell Transcriptome‐Wide Mendelian Randomization and Differentially Expressed Gene Analyses to Prioritize Dynamic Immune‐Related Drug Targets for Cancers

**DOI:** 10.1002/advs.202507451

**Published:** 2025-11-11

**Authors:** Jie Zheng, Qian Yang, Haoyu Liu, Huiling Zhao, Shuangyuan Wang, Yi Liu, Xueyan Wu, Yilan Ding, Hui Ying, Youqiu Ye, Xi Huang, Lei Ye, Ruizhi Zheng, Hong Lin, Mian Li, Tiange Wang, Zhiyun Zhao, Min Xu, Yi Duan, Hao Guo, Zhongshang Yuan, Philip Haycock, George Davey Smith, Richard M Martin, Guang Ning, Fang Hu, Weiqing Wang, Tom R. Gaunt, Jieli Lu, Yufang Bi

**Affiliations:** ^1^ Department of Endocrine and Metabolic Diseases Shanghai Institute of Endocrine and Metabolic Diseases Ruijin Hospital Shanghai Jiao Tong University School of Medicine Shanghai 200025 China; ^2^ Shanghai National Clinical Research Center for Metabolic Diseases Key Laboratory for Endocrine and Metabolic Diseases of the National Health Commission of the PR China Shanghai Key Laboratory for Endocrine Tumour Shanghai Digital Medicine Innovation Center Lifecycle Health Management Center, Ruijin Hospital Shanghai Jiao Tong University School of Medicine Shanghai 200025 China; ^3^ MRC Integrative Epidemiology Unit (IEU) Bristol Medical School University of Bristol Oakfield House, Oakfield Grove Bristol BS8 2BN UK; ^4^ Center for Immune‐Related Diseases at Shanghai Institute of Immunology Department of Gastroenterology Ruijin Hospital Shanghai Jiao Tong University School of Medicine Shanghai 200025 China; ^5^ Shanghai Institute of Immunology State Key Laboratory of Oncogenes and Related Genes Department of Immunology and Microbiology Shanghai Jiao Tong University School of Medicine Shanghai 200025 China; ^6^ Developmental and Stem Cell Biology Program The Hospital for Sick Children Toronto Ontario M5G 1X8 Canada; ^7^ Arthur and Sonia Labatt Brain Tumour Research Centre The Hospital for Sick Children Toronto Ontario M5G 1X8 Canada; ^8^ Department of Molecular Genetics University of Toronto Toronto Ontario M5S 1A8 Canada; ^9^ Key Laboratory of Immune Response and Immunotherapy Department of Life Sciences and Medicine University of Science and Technology of China Hefei Anhui 230026 China; ^10^ Department of Infectious Diseases The First Affiliated Hospital of USTC Department of Life Sciences and Medicine University of Science and Technology of China Hefei Anhui 230001 China; ^11^ State Key Laboratory of Cellular Stress Biology School of Life Sciences Faculty of Medicine and Life Sciences Xiamen University Xiamen Fujian 361102 China; ^12^ Xiang'an Hospital of Xiamen University School of Medicine Faculty of Medicine and Life Sciences Xiamen University Xiamen 361102 China; ^13^ Department of Biostatistics School of Public Health Cheeloo College of Medicine Shandong University Jinan 250012 China; ^14^ Population Health Sciences Bristol Medical School University of Bristol Bristol BS8 2BN UK; ^15^ NIHR Bristol Biomedical Research Centre, University Hospitals Bristol and Weston NHS Foundation Trust University of Bristol Bristol BS8 2BN UK; ^16^ National Clinical Research Center for Metabolic Diseases Key Laboratory of Diabetes Immunology Ministry of Education Department of Metabolism and Endocrinology the Second Xiangya Hospital of Central South University Changsha Hunan 410011 China

**Keywords:** cancer prevention, differential expression gene, drug targets, immune‐cell eQTLs, Mendelian randomization

## Abstract

Single‐cell expression quantitative trait loci data offer promising opportunities to inform immune‐related drug development in cancer. However, pleiotropy can complicate causal inference. We introduce MR‐DEG, a framework that integrates Mendelian randomization (MR) and differential expressed gene (DEG) to strengthen causal inference. Using eight conventional MR and colocalization methods, we estimated effects of 11 021 dynamic gene expression profiles during CD4+ T cell activation on the risk of six cancer types. This identified 1000 gene‐cancer pairs with putative effects (https://www.omicsharbour.com/sc‐eqtl‐mr). Of these 1000 pairs, 517 involved 205 unique genes that were differentially expressed in relevant cancer tissues based on single‐cell RNA‐sequencing data. Of these 517 pairs, 265 were classified as likely causal using the conventional MR methods. After applying MR‐DEG to the remaining 252 potentially pleiotropic pairs, an additional 89 were classified as likely causal. Sixty‐four and 391 of the 1000 original pairs exhibited time‐ and non‐time dependent effects on cancer risk, respectively. Integrating the 1000 gene‐cancer pairs of MR findings and clinical trial evidence, we identified 200 pairs corresponding to 33 unique genes that encode drug targets under clinical investigation. These results demonstrate how combining genetic, transcriptomic and clinical trial evidence can reduce pleiotropic bias, and prioritize immune‐related drug targets for cancer prevention.

## Graphical abstract

Inspired by evidence triangulation, a new sensitivity method called MR‐DEG  is developed, which uses the differentially expressed gene (DEG) results as additional evidence to minimize pleiotropic effects and strengthen Mendelian randomization (MR) causal estimates. Using dynamic single‐cell expression quantitative trait loci (eQTLs) as an example, it is demonstrated that MR‐DEG marked 35% of the MR estimates that showed potential pleiotropy as likely causal. By integrating MR‐DEG with genetic, genomic, biological, and trial evidence, eight target genes for cancer prevention are prioritized.

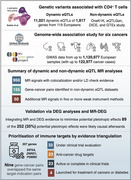

## 1 Introduction

The role of T cells in cancer has been recognized,^[^ ^1^ ^]^ including the elimination of tumor cells directly and indirectly.^[^ ^2^, ^3^ ^]^ Naïve T cells can be differentiated into a set of T helper (Th) cells and T regulatory cells (Tregs). The anti‐inflammatory and immune‐suppressive roles and antitumor immunity of Tregs have been widely recognized.^[^ ^3^ ^]^ T cells also play a central role in immune‐mediated drug development for cancers. Most of the successful immune‐mediated treatment approaches, including immune check point inhibitors, adoptive and chimeric antigen receptor T cell therapy, monoclonal antibodies, and therapeutic vaccination, are directed by T cell‐mediated anticancer responses,^[^ ^4^, ^5^, ^6^, ^7^ ^]^ and have shown considerable promise for the treatment of various solid cancer sites, including breast, lung and prostate.^[^ ^8^ ^]^ The two most notable immune‐mediated drug targets—cytotoxic T‐lymphocyte‐associated antigen 4 (CTLA4) and programmed death 1 (PD‐1)—both induce signals to prevent T cell activation.^[^ ^9^, ^10^ ^]^ Although strategies manipulating T cells have shown promise in anticancer drug development, most of the insights arose from animal models or a limited number of cancer patients.

Recently, many population‐based omics studies identified candidate drug target genes associated with complex diseases, including cancers.^[^ ^11^, ^12^, ^13^, ^14^, ^15^, ^16^, ^17^, ^18^, ^19^ ^]^ However, these studies used quantitative trait loci (QTLs) maps from bulk tissues, which represent mixed populations of cells rather than true single‐cell data, and thus represent the averaged expression signal over different types of cells. Such a setting limited the exploration of the heterogeneity of expression across individual cells, where cell type specificity has shown its value in predicting and guiding immune responses and identifying novel drug targets.^[^ ^20^ ^]^ Due to the recent development of single‐cell sequencing technology, several studies have identified expression QTLs (eQTLs) in various human immune cell types.^[^ ^21^, ^22^, ^23^ ^]^ Taking advantage of these single‐cell eQTL studies, we can prioritize immune‐mediated drug target genes at different activation stages. However, we also observed the following issues of single‐cell eQTL data: 1) the sample size of a single‐cell eQTL study was usually 10–100 times smaller than that of a bulk eQTL study, which limited the statistical power of identifying many eQTLs with good instrument strength; 2) given insufficient number of cells and RNA for each cell‐type, the sequencing depth of single‐cell RNA‐seq is lower compared to bulk RNA‐seq; 3) given the increased complexity of cell‐type‐related eQTLs, a single eQTL is more likely to be associated with expression of many genes at various cell‐types / activation time points. These create new challenges for downstream genetic analyses, especially in terms of potential weak instrument bias and pleiotropy.

Mendelian randomization (MR)^[^ ^24^ ^]^ and genetic colocalization^[^ ^25^ ^]^ are state‐of‐the‐art population genetics methods that can identify causal genes for complex diseases. These methods can benefit from the two‐sample setting, i.e., gene expression (exposure) and cancer (outcome) data from two different samples within the same underlying population,^[^ ^24^, ^26^ ^]^ which allows us to systematically estimate effects of altering expression of all available immune‐related genes on a range of cancer sites in one study.^[^ ^27^, ^28^ ^]^ We have previously applied this approach to identify immune‐related genes for cardio‐metabolic diseases.^[^ ^29^ ^]^ However, the existing pipeline was not designed to deal with the abovementioned challenges raised by single‐cell eQTL data. Novel methodological attempts are needed to better utilize these new data to infer causality.

In addition, differentially expressed gene (DEG) analysis is a classic approach to evaluate the associations between gene expression and a disease,^[^ ^30^ ^]^ especially for cancer.^[^ ^31^ ^]^ This approach relies on different sources of data and methodology compared with MR. However, both approaches aim to address a very similar scientific question—whether specific genes are implicated in risk of disease. When findings from these independent methods converge, they provide evidence triangulation, meaning that results from complementary analytical frameworks mutually corroborate each other and thereby strengthen causal inference.^[^ ^32^, ^33^ ^]^ In addition to the increase in reliability through external validation, integration of evidence between the two state‐of‐the‐art methods may address biases that could not be solely solved by either of them.

**Figure 1:**
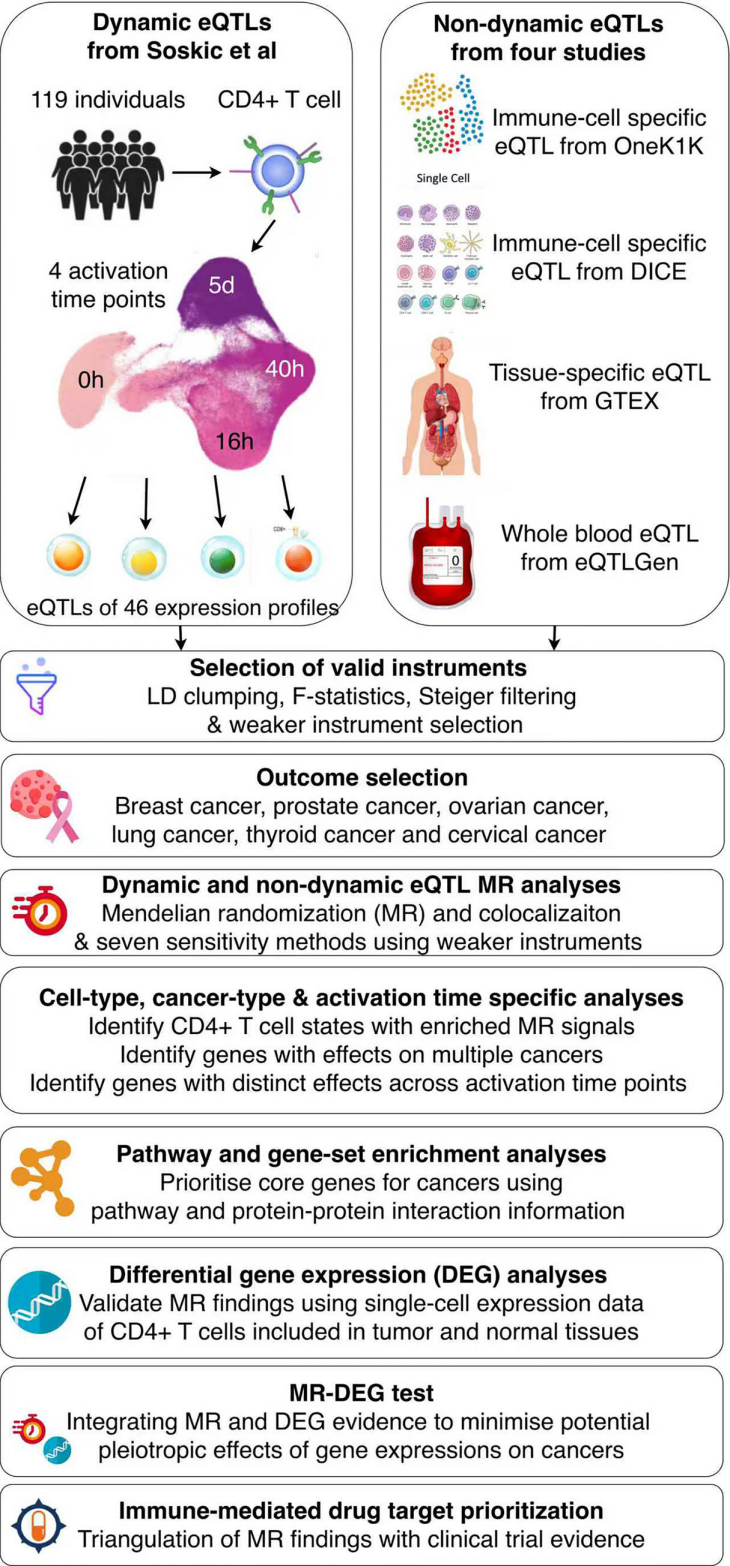
Study design of the current study. Abbreviations: eQTLs, expression quantitative trait loci; LD, linkage disequilibrium.

In this study, we systematically estimated the effects of 11 021 dynamic gene expression profiles (all termiologies listed in **Table** **1**) during CD4+ T cell activation^[^ ^21^ ^]^ on six common cancers by developing a new MR pipeline (see **Figure** **1**). Our pipeline paid special attention to challenges raised by single‐cell eQTL data. In this study, an expression profile was defined as a gene‐cell type‐activation time point combination (Table 1). First, a weak instrument selection pipeline together with seven methods that could deal with weak instrument bias was applied as validation of the main MR analysis using strong instruments. The comparison MR analysis was further conducted using data from four non‐dynamic eQTL datasets^[^ ^12^, ^22^, ^23^, ^34^ ^]^ to investigate whether dynamic single‐cell type eQTL MR can support the identification of novel cancer‐linked genes. The DEG analysis was applied for two purposes: 1) to externally validate the MR findings in cancer cells; 2) to propose a new method, “MR‐DEG ”, which provides additional evidence to minimize the influence of pleiotropy on the single‐cell MR findings. We further estimated the cell‐type, activation time, and cancer‐type‐specific effects on the gene‐cancer associations, applied pathway and gene‐set enrichment analyses to prioritize core genes, and integrated MR findings with clinical trial evidence to prioritize immune‐related targets for cancers. Using our heuristic approach to highlight estimated effects worthy of follow‐up, we report our results in an openly accessible database, Omics Harbour single‐cell eQTL MR browser (https://www.omicsharbour.com/sc‐eqtl‐mr).

**Table 1:** Definition of related terminologies in this study.

Term	Definition
Expression profile	A global pattern of gene expression defined by cell type, condition, and time point.
eGene	A gene with cis‐expression quantitative trait loci (eQTL) associated with the expression levels of the relevant gene reported by Soskic et al.
Dynamic eQTL	A genetic variant associated with the expression level of a gene during CD4+ T cell activation.
Gene‐cancer pair	A gene‐cancer combination, regardless of the gene expression profiles, e.g., *FADS1*‐lung cancer.
Cancer‐linked genes	Genes that showed Mendelian randomization and colocalization evidence to support their causal effect on cancers.
Target‐indication pair	A combination of one drug and one indication of the drug, information derived from a clinical trial.

We clarified the focus of this study. First, the single cell eQTL data were collected from whole blood samples from the general population, rather than individuals with cancers. This is because stratifying the exposure (i.e., expression of a gene) into subgroups (e.g., cancer cases) can lead to collider bias,^[^ ^35^ ^]^ which could break the randomization and induce spurious associations between the target genes and cancers.^[^ ^36^ ^]^ Second, genome‐wide association studies (GWAS) of cancers are based on incident/prevalent cancer cases in a case‐control setting. Therefore, our study focused on investigating possible immune‐mediated targets for cancer prevention, rather than cancer treatment.

## 2 Results

### 2.1 Methods Summary

#### 2.1.1 Selection of Genetic Instruments and Cancer Outcomes

Our study investigated the dynamics of gene expression during activation of naïve and memory CD4+ T cells and their sub‐populations (including central memory (TCM), effector memory (TEM), effector memory cells re‐expressing CD45RA (TEMRA), and regulatory T cells (Treg).

For the main MR analysis, 11 433 conditionally independent dynamic *cis*‐eQTLs (genetic variants associated with the regulation of gene expression dynamics throughout CD4+ T cell activation in the *cis*‐acting regions) of 1823 genes expressed in 46 expression profiles (all termiologies listed in Table 1) were selected as candidate genetic instruments for the respective genes (Figure 1). These 46 expression profiles included genes expressed in 19 cell‐types (CD4 naïve, TN [Native T cell], TN cycling, TN HSP [heat shock protein], TN IFN [interferon], TN LA [lowly active], TN NFKB [nuclear factor κB], HSP, CD4 memory, TEM, TCM, TEM HLA positive, TEMRA, TEM LA, TCM LA, nTreg [natural regulatory T cell], TM cycling, TM ER‐stress [endoplasmic reticulum], T ER‐stress) during five cell activation time points (0 h, lowly active [LA], 16 h, 40 h and 5 d). The data were derived from single‐cell sequencing of 119 Europeans.^[^ ^21^ ^]^ To increase the reliability of the instruments, we applied an instrument validation pipeline to filter eQTLs that best fit the MR assumptions (more details in Online Methods). After selection, 11 021 cis‐eQTLs of 1817 genes in 46 CD4+ T cell expression profiles were selected as instruments for the MR analysis (Table S1A, Supporting Information).

Given the sequencing depth of single‐cell RNA‐seq, we were only able to identify a small number of strong instruments per exposure (cell‐type and time point‐specific gene expression profiles), which limited our ability to identify true causal effects. Therefore, we used a more relaxed genetic association *p*‐value threshold (*p* < 0.05) and *F*‐statistic (≥ 5) to select instruments. After applying this process with Steiger filtering and LD clumping (LD r^2^ < 0.1 referring to the 1000 Genome Project Europeans), we identified 11 007 of the 11 021 expression profiles with valid instruments (47 051 instruments), of which 845 of the profiles only have one instrument, with the remaining 10 162 profiles having two or more instruments (Table S1B, Supporting Information).

We further compared the top dynamic CD4+ T cell eQTL MR findings (which passed a Benjamini‐Hochberg false discovery rate [FDR]^[^ ^37^ ^]^ correction threshold of 0.05) with MR results obtained from other non‐dynamic eQTL datasets with different properties: immune cell eQTLs from OneK1K^[^ ^23^ ^]^ and DICE consortia,^[^ ^22^ ^]^ as well as tissue‐specific eQTLs from GTEX^[^ ^34^ ^]^ and eQTLGen^[^ ^12^ ^]^ consortia (Figure 1). After instrument selection, 6318 instruments for 392 genes (5227 CD4+ T cell expression profiles) were selected to compare with the top findings identified from the dynamic eQTL MR analysis (Table S1C–F, Supporting Information).

For the outcomes of the dynamic and non‐dynamic eQTL MR analyses, we selected five cancers from the IEU OpenGWAS database^[^ ^38^, ^39^, ^40^, ^41^, ^42^ ^]^ and thyroid cancer from the Global Biobank Meta‐analysis Initiative (GBMI)^[^ ^43^ ^]^ on the basis that we had full GWAS summary statistics and relatively good statistical power (more than 1000 cases; Table S2, Supporting Information).

#### 2.1.2 Summary of Dynamic and Non‐Dynamic eQTL MR Analyses

We undertook two (dynamic and non‐dynamic eQTL) MR and sensitivity analyses to systematically evaluate evidence for the associations of expression levels of 1817 genes expressed at different cell states, activation time points, and tissues (11 021 exposures for dynamic eQTL MR analysis and 5227 exposures for non‐dynamic eQTL MR analysis) with the six cancers. We applied the Wald ratio test^[^ ^44^ ^]^ as the main MR analysis, and only included those with exactly the same SNPs in the outcome data, and did not search for any proxy SNP. As a test of the influence of missing data, we searched for proxy genetic variants in high linkage disequilibrium (LD) with the eQTL (r^2^>0.9 in the 1000 Genomes data for the relevant population^[^ ^45^ ^]^) in the outcome data instead (Figure 1).

For dynamic eQTL MR analysis using weaker instruments, we applied Wald ratio and seven additional MR methods, including weighted median, weighted mode, MR‐Robust, cML‐MA, MR‐PRESSO (which needs four or more instruments), debiased IVW and MR‐RAPS, to estimate the effect of 11 007 exposures on six cancer sites, aiming to compare the performance of these methods in terms of single‐cell QTL MR analysis.

#### 2.1.3 Estimated Effects of Dynamic Gene Expression in CD4^+^ T Cells on Cancers

For the main MR of 1817 genes at different cell‐types and time‐points against six cancers, the effects of 48 630 gene‐cancer pairs were tested, and all pairs passed the MR Steiger filtering test, suggesting that reverse causality is unlikely to be a major issue for this study. Among these pairs, 994 MR signals reached an FDR threshold of 0.05 (Table S3, Supporting Information).

**Figure 2:**
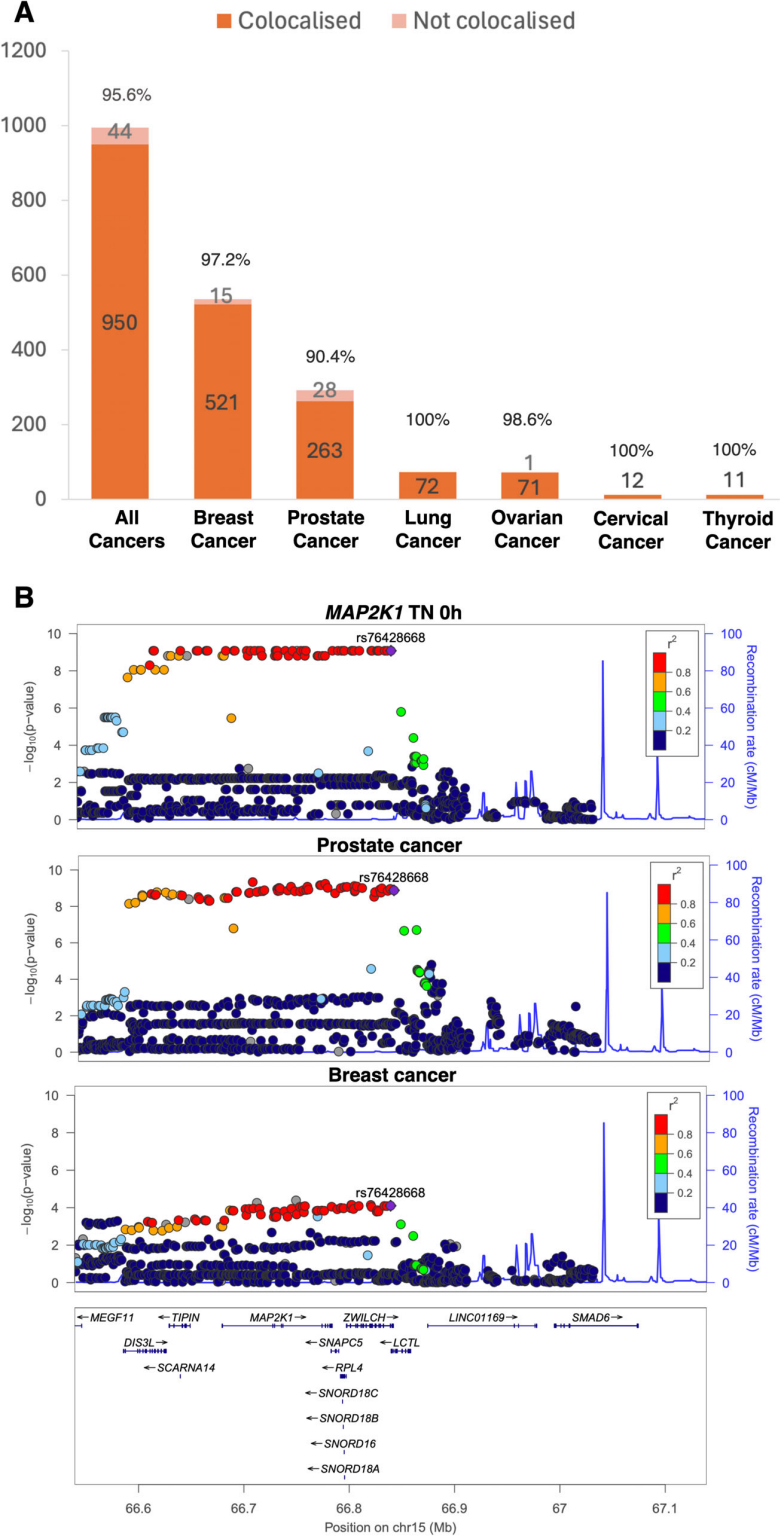
Summary of main Mendelian randomization (MR) findings. A) percentage of top MR findings with colocalization evidence. B) regional plots of *MAP2K1* expression (naïve T cell [TN] at resting stage [0 h]), prostate cancer and breast cancer in the cis *MAP2K1* genomic region.

To distinguish putative causal gene‐cancer pairs from confounding by LD, we applied two colocalization approaches on the top MR signals that passed the FDR threshold: genetic colocalization^[^ ^25^ ^]^ and LD check analysis.^[^ ^13^ ^]^ The LD check analysis, which estimated the LD between each eQTL and cancer‐linked genetic association signals in the cis region, suggested that 895 MR signals (for 850 exposures of 225 gene‐cancer pairs) showed evidence of approximate colocalization (pair‐wise LD r^2^>0.7; Table S4A, Supporting Information). The colocalization analysis provided evidence for 519 MR signals of 133 gene‐cancer pairs (colocalization posterior probability PP.H4>70%). In summary, 950 of 994 (96%) MR signals showed colocalization and/or LD check evidence, in which the proportion of colocalization was from 90% to 100% for different cancer types (**Figure** **2**A). For example, we identified a positive effect of gene expression level of *CALR* in TN 40 h on breast cancer (odds ratio [OR] = 0.94, 95% confidence interval [CI] = 0.90 to 0.97, *p* = 3.4 × 10^−4^, colocalization probability PP.H4 = 94%; Table S4A, Supporting Information). The related drug, vasostatin, has been under pre‐clinical development as an anticancer drug.^[^ ^46^ ^]^

Among the associated genes for cancer, 19 genes showed robust MR and colocalization evidence on two cancer sites (Table S4A, Supporting Information). As an example of genes with MR and colocalization evidence on two cancer sites, *MAP2K1* in cell type TN at resting (0 h) showed colocalization evidence on prostate cancer and breast cancer (colocalization probability PP.H4 = 94% and 93% respectively; Figure 2B). The drug targeting *MAP2K1*, Binimetinib, had been launched for cancer treatment (NCT01556568). Although our approach was set up to identify targets for cancer risk, given that there is an existing anticancer drug for this target, we are reasonably confident that the effect of these drugs on prostate cancer merits further investigation for a repurposing opportunity in future trials. The MR results identified using LD proxy search were listed in Table S4B (Supporting Information).

We further conducted MR of the 11 007 exposures (Table S1B, Supporting Information) on six cancer sites using weaker instruments and eight MR methods. Among the 950 main MR signals with colocalization evidence, 95% of them could be validated in at least one of the other methods using weaker instruments. Given the complex LD of the major histocompatibility complex (MHC) region in chromosome six, we paid specific attention to this region and found that 84% of the main MR findings could be validated by other methods. Excluding genes within this region, 97% of the main MR findings could be validated by other methods.

In addition, 50 MR signals showed robust MR evidence (FDR corrected *p*‐value<0.05) in five or more weak instrument MR methods, but did not show strong MR evidence in the main MR analysis (Table S4C, Supporting Information), which showcased the ability of weaker instruments in identifying novel gene expression‐cancer associations. In total, 1000 MR signals were identified by using dynamic single‐cell eQTL data, which included 50 signals from weak instrument analysis and 950 from the main MR analysis using strong instruments.

To support future single‐cell eQTL MR studies, we compared the power and robustness of the eight MR approaches using weak instruments. This comparison showed that MR‐Robust is the most powerful method when using weak instruments, with 6% of MR estimates passing FDR‐corrected p‐value cutoff, followed by MR‐PRESSO (4%) and cML‐MA (2%; Table S4D, Supporting Information). The robustness test showed that debiased IVW, weighted mode, and weighted median findings could be 100% validated in two or more other methods, followed by MR‐RAPS (76%) and MR‐Robust (70%; Table S4D, Supporting Information). Considering both power and robustness, we recommended MR‐Robust as the discovery approach for weak instrument single‐cell eQTL MR, and weighted median, debiased IVW, and MR‐RAPS as reliable validation methods.

An important assumption of our study is that gene expression of T cells in blood, proxied by blood‐based eQTLs are relatively similar to gene expression of T cells in solid tumor cells, proxied by tumor tissue‐based eQTL. We conducted three analyses to test this assumption. First, an analysis comparing beta coefficients of whole blood eQTLs versus tissue‐specific eQTLs showed very high correlation (Pearson correlation [r] >0.82 for all five tested tissues; Figure S1 (Supporting Information); data from tissue‐specific eQTLs from GTEX v10). A second validation analysis using single‐cell RNA‐seq data from paired blood–tumor samples showed that CD4+ T cell expression in blood and in tumor cells also had a high correlation (r = 0.93; Figure S2, Supporting Information). Third, we compared the MR estimates using CD4+ T cell eQTLs from blood as instruments versus those using tumor‐tissue‐based eQTLs as instruments (data from PancanQTL database). Among the unique gene‐cancer pairs with top MR evidence from the main MR analysis (Table S4A, Supporting Information) and with available eQTL instruments from PancanQTL database, 48% of them showed the same direction of effect and were marginally associated with the outcomes in MR (MR *p* < 0.05) in PancanQTLs MR estimates (Table S4E, Supporting Information). These results supported our assumption that we can consider using eQTLs in blood to proxy eQTLs in tumor cells to some extent.

To validate whether our approach could be extended to other cancer sites, we conducted an MR analysis of the CD4+ T cell gene expression exposures across pan‐cancer. Both strong and weak instruments approaches were applied. The pan‐cancer outcome GWAS was derived from FinnGen plus UK Biobank (N cases = 187 278, N controls = 722 095). This analysis identified 522 genes whose expression during CD4+ T cell activation has potential effects on pan‐cancer risk using a strong instrument approach and identified 23 additional effects that were validated using five or more weak instrument methods (Table S4F, Supporting Information). This analysis showed that our approach was able to identify cancer‐linked genes on a pan‐cancer scale.

#### 2.1.4 Overlap with Non‐Dynamic Tissue‐ and Cell‐Type Specific eQTL MR Estimates

For the comparison MR using additional non‐dynamic eQTL data from four studies,^[^ ^12^, ^22^, ^23^, ^34^ ^]^ 155 unique gene‐cancer pairs (which is a “unique gene‐disease pair,” a pair regardless of the gene expression profiles, e.g., *FADS1*‐lung cancer) identified in the dynamic eQTL MR analysis also showed MR and colocalization evidence using the non‐dynamic eQTL datasets (Tables S5–S8, Supporting Information). In more detail, 7 of 8 pairs colocalized using OneK1K data (Table S5, Supporting Information), 115 of 138 pairs colocalized using eQTLGen data (Table S6, Supporting Information), 320 of 522 pairs colocalized using DICE data (Table S7, Supporting Information), 1927 of 3453 pairs colocalized using GTEX data (Table S8, Supporting Information).

**Figure 3:**
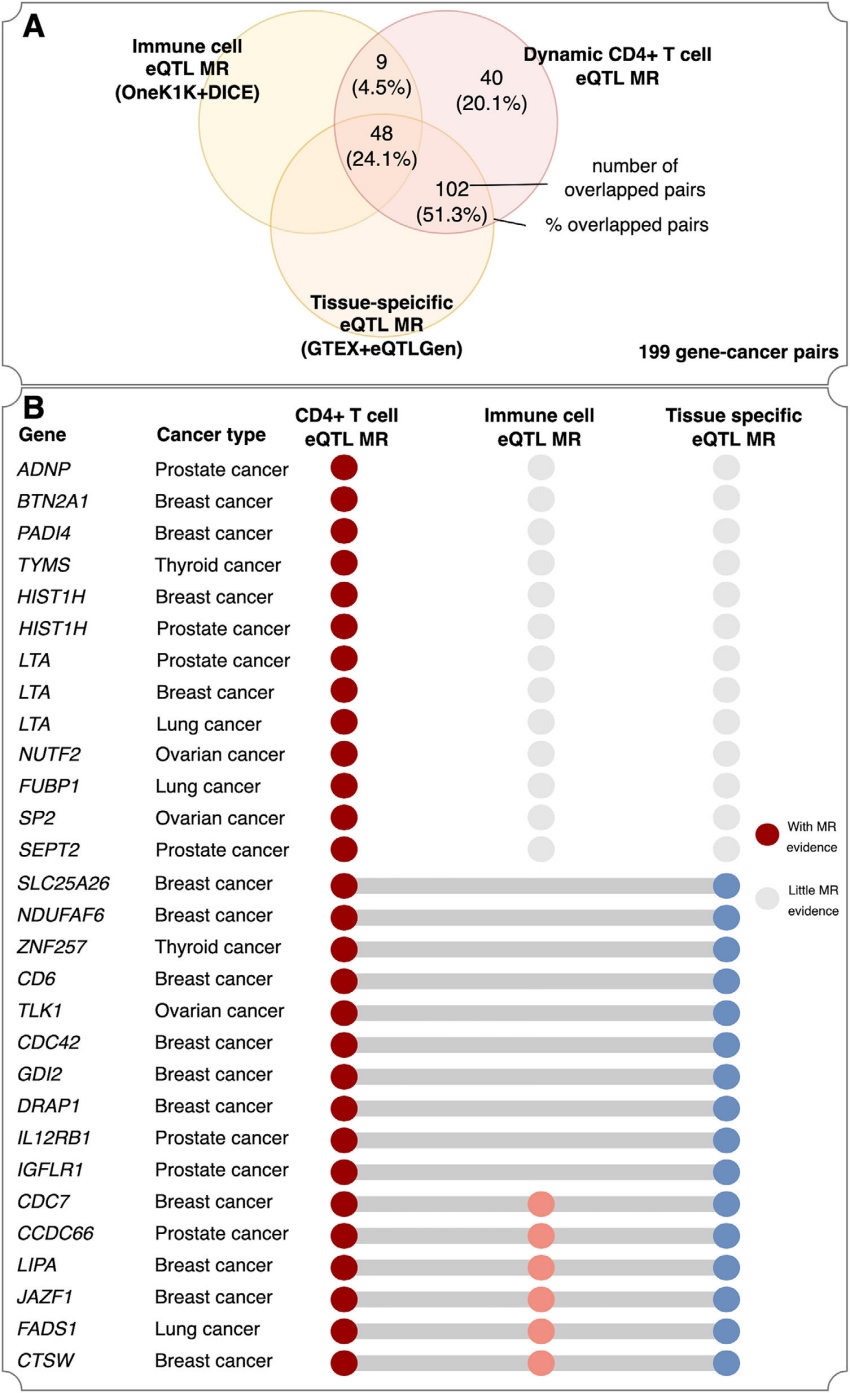
Validation Mendelian randomization (MR) results. A) percentage of dynamic expression quantitative trait loci (eQTL) MR top findings identified by non‐dynamic eQTL MR using instruments from four other eQTL studies. The other four eQTL studies were OneK1K, a single‐cell eQTL study of immune cells; DICE, an immune cell eQTL study using bulk tissue plus imputation of expression; and GTEX, a tissue‐specific eQTL study of 49 human tissues (more details of these studies were described in Online Methods). B) Selected MR findings that have been detected using different eQTL datasets.

In a systematic comparison between the dynamic and non‐dynamic eQTL MR findings, we observed that 67 of 224 (30%) unique gene‐cancer pairs only showed robust MR and colocalization evidence using dynamic CD4+ T cell eQTLs, but not in any other non‐dynamic eQTL datasets (**Figure** **3**A). For example, the expression of *PADI4* in TN 16 h showed an effect on breast cancer (OR = 1.03, 95%CI = 1.01 to 1.05, *p* = 2.7 × 10^−3^). In contrast, although with good instruments in other eQTL datasets, *PADI4* expression showed little MR evidence on breast cancer using other eQTL data (Figure 3B; Tables S4–S8, Supporting Information).

#### 2.1.5 Differentially Expressed Gene Analysis of Prioritized Genes in CD4+ T Cells in Cancer Tissues

We used gene expression data of CD4+ T cells derived from whole blood as exposures to identify the cancer‐linked genes. Although previous studies showed that whole blood transcriptome can predict the tissue‐specific expression levels for approximately 60% of the genes across various tissues,^[^ ^47^ ^]^ we still tested cell‐type‐specific effects in the cancer micro‐environment. For the 224 unique gene‐cancer pairs with robust MR and colocalization evidence, we conducted a differentially expressed gene (DEG) analysis^[^ ^48^ ^]^ as independent external validation. To better mimic the cell‐type setting of the MR analyses, single‐cell gene expression data of conventional T cells (Tconvs), derived from normal and tumor tissues from 16 studies,^[^ ^49^, ^50^, ^51^, ^52^, ^53^, ^54^, ^55^, ^56^, ^57^, ^58^, ^59^, ^60^, ^61^, ^62^, ^63^, ^64^ ^]^ were used for the DEG analysis. The results suggested that 517 (51.7%; 500 from strong instrument analysis, 17 from weak instrument analysis) of the 1000 gene‐cancer MR signals were externally validated by the DEG analysis (*p* of DEG < 0.05 and the logFC value showed the same direction of effect as the MR estimate; Table S4A and S4C, Supporting Information). These 517 pairs refer to 205 unique genes aganist six cancer types (Table S4G, Supporting information).

#### 2.1.6 MR‐DEG Minimize Potential Pleiotropy for the Prioritized Genes

Given that many more exposures have been created by single‐cell RNA‐seq (e.g., one exposure for bulk RNA‐seq versus 46 exposures for single‐cell RNA‐seq in Soskic et al.^[^ ^21^ ^]^), single‐cell eQTLs suffer more serious issues of pleiotropy compared to bulk eQTLs. For the 11 021 single‐cell eQTLs instruments we used, each eQTL was on average associated with seven exposures when using 1×10^−4^ as the association *p*‐value cutoff. Given that we have a limited number of instruments for each exposure, it is almost impossible to exclude potential pleiotropy solely using existing statistical genetics methods. Inspired by the concept of evidence triangulation,^[^ ^32^ ^]^ we found that single‐cell DEG analysis aims to identify the association between gene expression in a cell type and an outcome (e.g., a type of cancer), so as to answer a similar scientific question to a single‐cell eQTL MR analysis but based on an independent source of data and different methods. In addition to being used as an external validation, we consider that single‐cell DEG evidence could be used to minimize potential pleiotropy. Specifically, if a genetic variant is associated with the expression of multiple genes (A and B) or the expression of a gene in multiple cells (gene A in cell types 1 and 2), this is known as pleiotropy in human genetics. In such a case, if the DEG analysis did not support the association of expression of gene A in cell type 2 (or expression of gene B) with the outcome, but showed a robust association with expression of gene A in cell type 1, this increases the possibility to support the expression of gene A in cell type 1 as a putative causal gene‐cell‐type combination and removes the potential pleiotropy effect for other genes and cell types (see Figure S3, Supporting Information). Given its property, we noted this concept as MR‐DEG. Among the 1000 gene‐cancer pairs, 517 of them showed MR, colocalization and DEG evidence. 265 of the 517 pairs were classified as likely causal since their instruments were only associated with one gene expression profile. For the remaining 252 potential MR signals, we applied MR‐DEG tool that also showed potential pleiotropy (instruments associated with two or more exposures with *p* < 1×10^−4^, Table S4A, Supporting Information). We found that 89 of the 252 (35%) potential pleiotropic effects were attenuated and considered as likely causal after applying the tool.

#### 2.1.7 Cell‐Type, Cancer‐Type, and Activation Time Specific Effects of CD4+ T Cell Expression on Cancers

##### Cell‐Type Specific Effects of Gene Expression of CD4+ T Cell on Cancers

**Figure 4:**
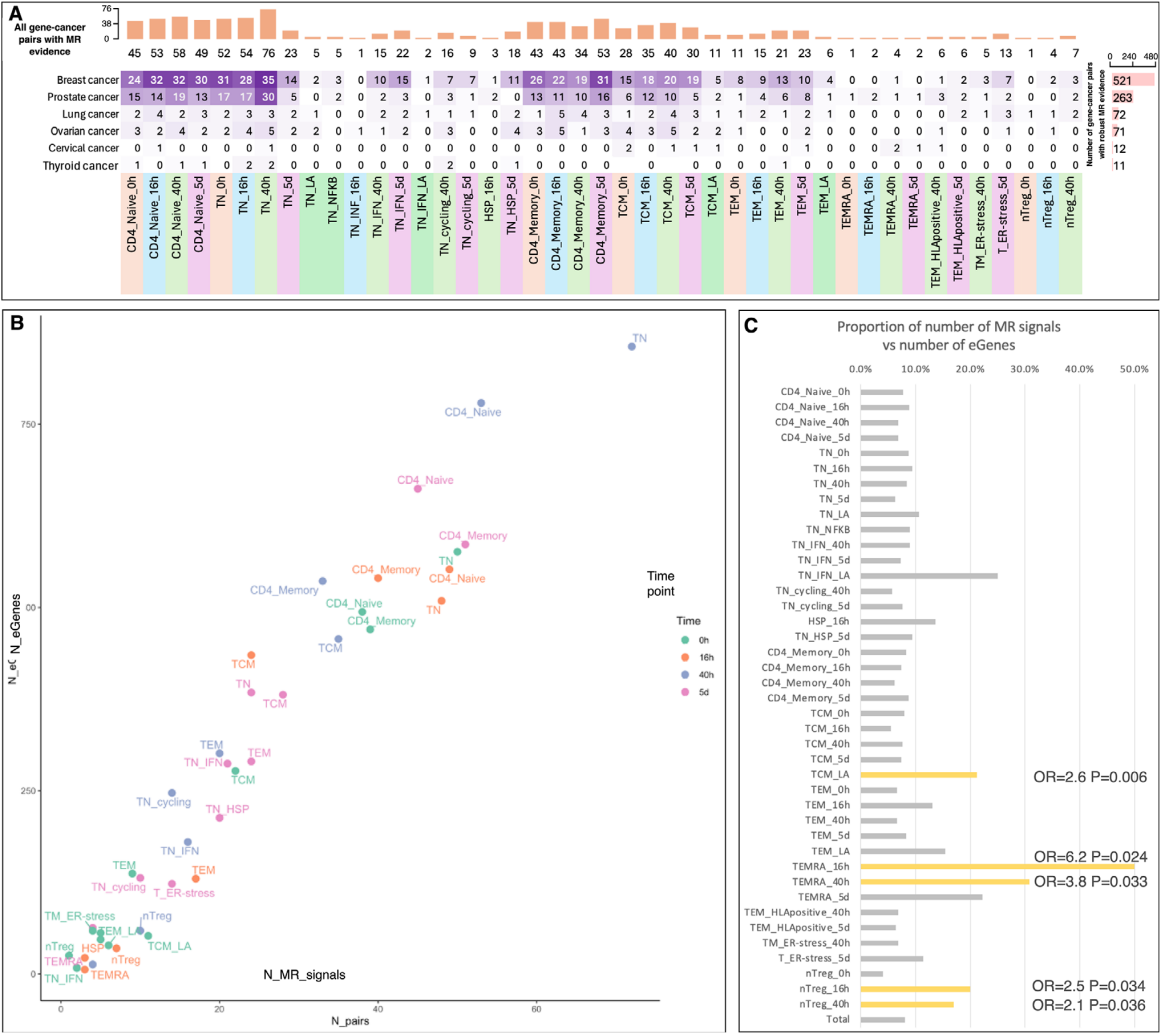
Cell‐type specific Mendelian randomization (MR) findings. A) Gene‐cancer pairs with Mendelian randomization and colocalization evidence stratified by cell types, dynamic change status and cancer types. B) Number of eGenes with instruments versus number of MR results for each cell‐type. One point is a gene‐cancer pair in one cell state/time point combination. Each color refers to one activation time point. C) proportion of top MR signals versus number of eGenes of the same cell‐type/time point combination, which indicated the level of enriched MR signals of each cell‐type after controlling the influence of number of eGenes.

For the 1000 gene‐cancer pairs that showed robust genetic evidence in the dynamic eQTL MR analysis, we illustrated the distribution of gene‐cancer pairs across different cell types, time points, and cancer types (**Figure** **4**A). First, we observed a very high correlation between the number of eGenes (Table 1) versus the number of MR signals (Spearman correlation = 0.96; Figure 4B), which showed that the more eGenes for a certain cell‐type/activation time point, the more MR signals we can identify in that cell‐type. After controlling the influence of the number of eGenes of each cell‐type, Fisher's exact test suggested that TEMRA (16 h, 40 h), TCM_LA, and nTreg (16 h, 40 h) had enriched MR signals compared to other cell‐types (Figure 4C). We then compared cell‐type specificity of naïve against memory CD4+ T cells for all 255 gene‐cancer pairs with robust MR and colocalization evidence in either naïve or memory CD4+ T cells (Table S9A, Supporting Information). The results showcased the distinct effects of the same genes across naïve and memory CD4+ T cells (Figure S4, Supporting Information).

We further tested the immune cell‐type specificity of the 207 identified unique cancer‐linked genes using cell‐type‐specific RNA‐seq data from the DICE consortium.^[^ ^22^ ^]^ We found that 25 of the cancer‐linked genes (12%) showed T cell specificity (expression levels of a gene in T cells were at least two times higher than expression levels the same gene in other immune cell types; Table S9B, Supporting Information), where expression levels of three genes, *SYNM*, *NIPAL1* and *TMEM45B*, showed CD4 + T cell specificity.

##### Cancer‐Type Specific Effects of Gene Expression of CD4+ T Cells on Cancers

For cancer‐type enrichment, more gene‐cancer pairs were observed for breast cancer and prostate cancer due to the greater power of the outcome GWAS data (Figure 4A). Balancing out the influence of sample size (number of cases), we found that genes expressed in CD4+ T cells were more likely to affect risk of lung cancer (OR of Fisher's exact test = 1.7, *p* = 3.66 × 10^−5^) and cervical cancer (OR = 4.7, *p* = 4.12 × 10^−12^).

We further estimated whether the identified cancer‐linked genes are specific to one cancer site or multiple cancer sites. This analysis suggested that 43% of the CD4 T cell‐related genes may not be specific to one cancer type (Table S10, Supporting Information), but we need more powerful GWAS data to validate the robustness of these marginal MR findings with weaker genetic signals.

##### Temporal Effects of Gene Expression of CD4+ T Cell on Cancers

**Figure 5:**
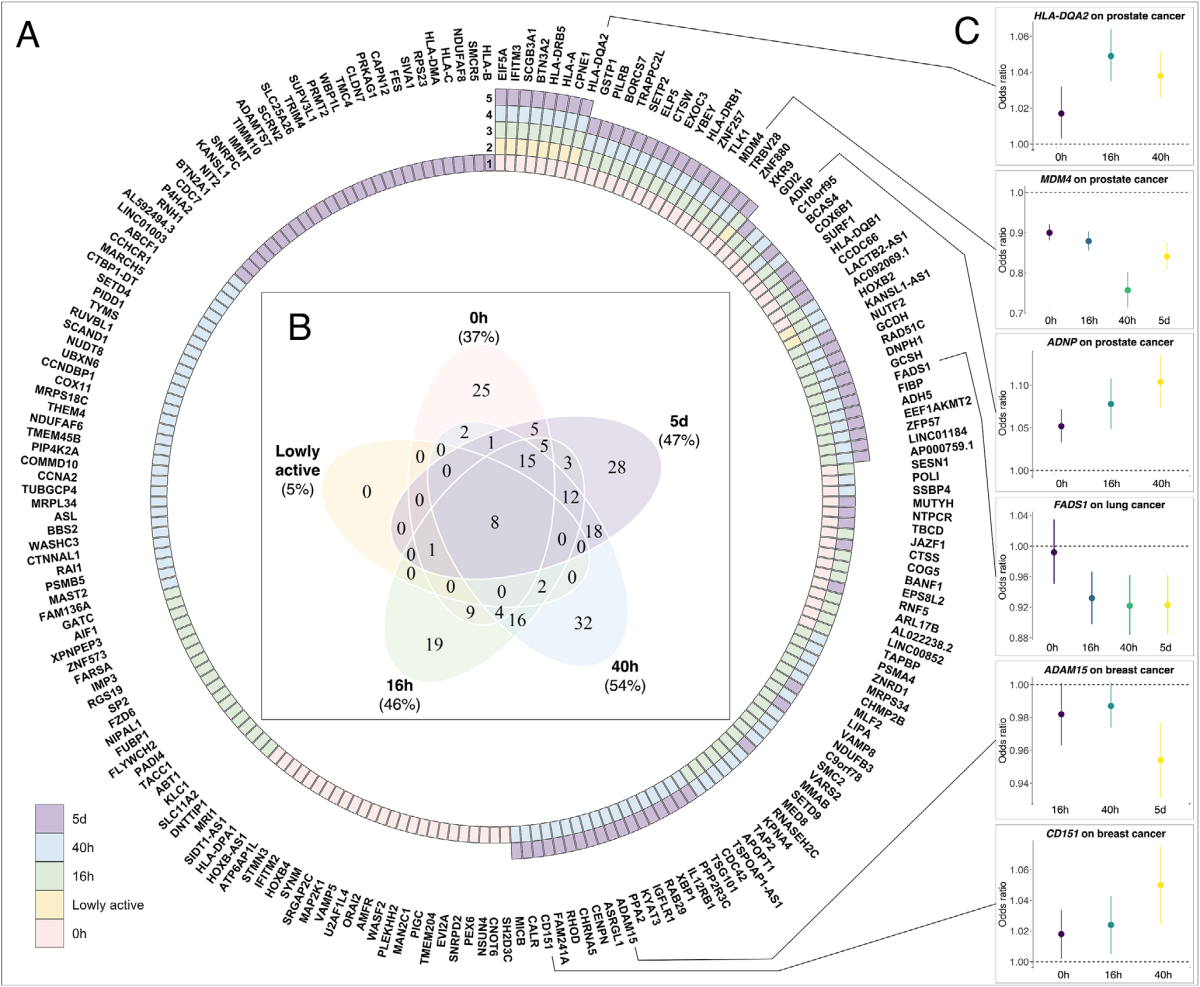
Activation time‐dependent effect of gene expression on cancers. A) the effect of expression levels of 207 cancer‐linked genes on cancers at five activation time points; B) proportion of cancer‐linked genes that showed up in five activation time points; C) six specific gene‐cancer pairs that showed distinguish causal effects across activation time points.

The effects of gene expression levels across multiple activation time points on cancers have not been temporally explored before. Here, we investigated this by utilizing the unique dynamic single‐cell eQTL datasets from Soskic et al, which have eQTLs of CD4+ T cell at five different activation time points (resting [0 h], lowly active [LA], before dividing [16 h], after the first cell division [40 h] and after acquiring effector functions [5 d].^[^ ^65^ ^]^ Of 207 cancer‐linked genes, 75 (37%) were identified in the resting state [0 h], but 130 (63%) of the identified genes were only detectable after T cell activation (**Figure** **5**A,B). Among 624 gene‐cell‐type‐cancer triplets, 213 (34%) of the identified genes showed effects on one cancer type at two or more time points (Table S4A, Supporting Information).

Furthermore, we aimed to systematically identify gene‐cancer pairs with distinct MR signals across activation time points. Among all 48 630 gene‐cancer pairs that have been tested, we extracted 740 MR results of 455 gene‐cancer pairs with robust MR signal (FDR<0.05) in one activation time point and with MR estimates (regardless of the FDR threshold) in other time points (Table S11A, Supporting Information). For each of the 455 pairs, the time point with largest sum of the absolute differences between each pairs of MR estimates was set as the reference, pair‐wise Z score was estimated between the reference MR effect and the effect at another time point (i.e., MR effects of expression of *ADAM15* in TCM at 40 h on breast cancer [set as the reference] were tested against that at 16 h and 5 d respectively). 64 unique gene‐cell‐type‐cancer triplets had robust MR signals in one (or more) time point and distinguished effects at other time points (*p*value of Z < 0.05; Table S11B, Supporting Information)，which implied time‐dependent effects. For instance, expression levels of *FADS1* in memory T cells showed little evidence to support an effect on lung cancer at 0 h but showed much stronger effects at 16 h, 40 h, and 5 d (Figure 5C). Among the 64 pairs with activation time‐specific MR effects, eight of them were drug targets that are currently under clinical trials (Figure 5C). In addition, there were 391 unique gene‐cancer pairs that showed no time‐dependent effects (Table S11B). This highlighted the value of considering the temporal pattern of effects of cancer‐associated genes in the drug development pipeline.

##### Pathway and Gene‐Set Analyses of the Cancer‐Linked Genes

Among the 207 cancer‐linked genes with robust MR and colocalization evidence, we further conducted a set of pathway and gene‐set enrichment analyses, aiming at identifying core genes and biological pathways that are representative of the identified cancer‐linked genes.

**Figure 6:**
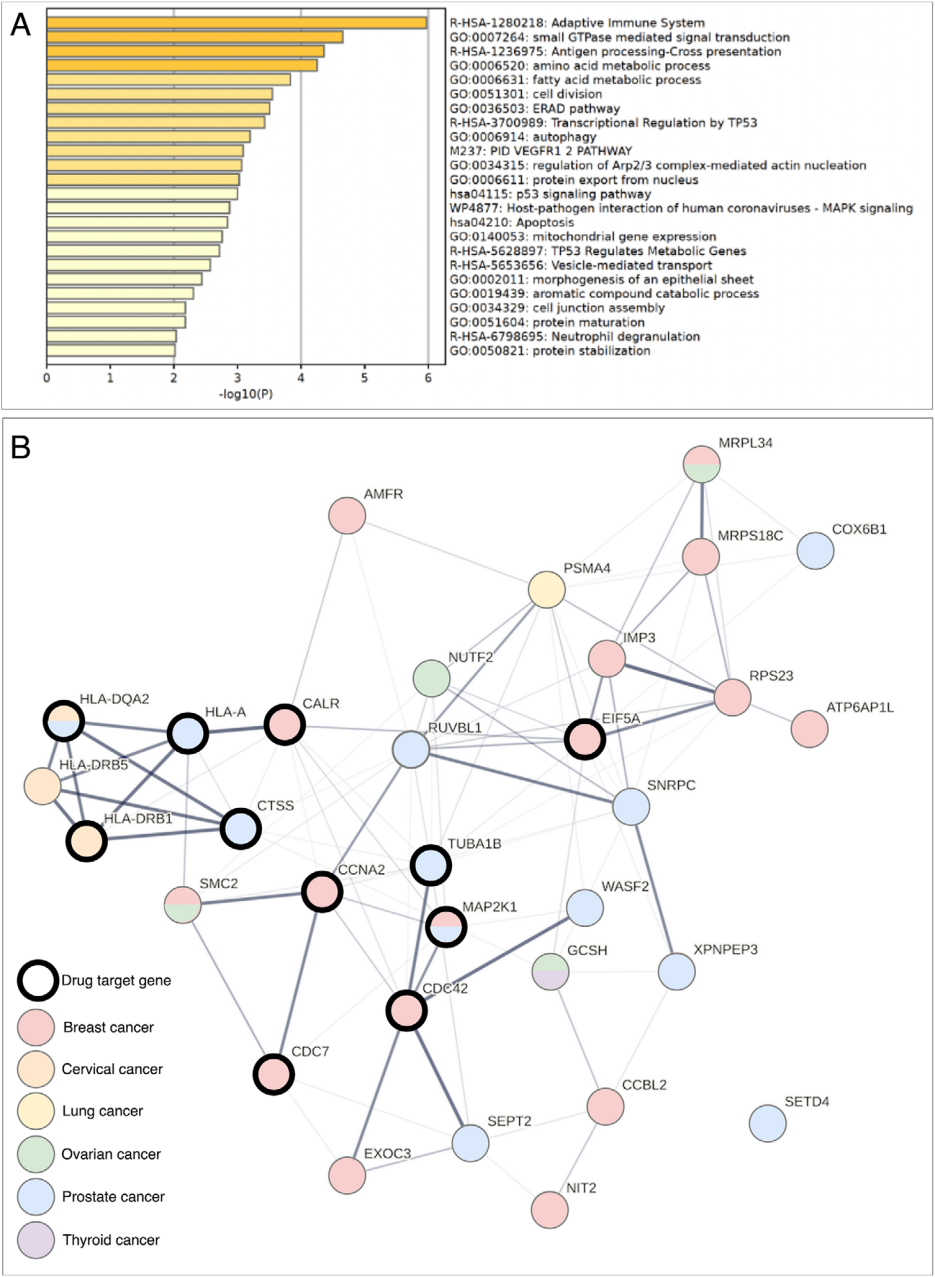
Pathway and gene‐set enrichment analyses results of the 27 core genes. A) Bar plot for pathway enrichment using Metascape. B) Protein‐protein interaction network and core genes identified in each cancer type. Genes highlighted in black were genes under clinical trial investigation for cancer/immune‐related disease treatment. Genes with multiple colors were those affecting risk of two or more cancers.

The pathway enrichment analysis using Metascape^[^ ^66^ ^]^ showed that the 207 cancer‐linked genes were enriched on 20 pathways, including the antigen processing and presentation of peptide antigen and the mitogen‐activated protein kinase pathway (**Figure** **6**A), known pathways that play important roles in cancers and chemotherapy.^[^ ^67^, ^68^ ^]^ Pathway analysis using Reactome^[^ ^69^ ^]^ identified 19 pathways, including immunotherapy related pathways such as PD‐1 signaling^[^ ^70^, ^71^ ^]^ (Table S12, Supporting Information).

The protein–protein interaction (PPI) network of the 207 cancer‐linked genes was created using data from StringDB database.^[^ ^72^, ^73^ ^]^ The core genes for the six cancers and each cancer type were estimated using the Cytoscape (version 3.6.1)^[^ ^74^ ^]^ plugin cytoHubba, respectively.^[^ ^75^ ^]^ As shown in Figure 6B, 27 cancer‐linked genes were identified as core genes (with maximal clique centrality [MCC] ≥ 9, a parameter to discover featured nodes from a PPI network) in the gene‐set enrichment analysis (Table S13A, Supporting Information). Two groups of genes within the 27 genes were clustered using MCODE plugin (Figure S5, Supporting Information): cluster 1 with 10 genes: *HLA‐A*, *HLA‐DQA2*, *HLA‐DQB1*, *HLA‐DMA*, *HLA‐C*, *HLA‐DRB5*, *HLA‐B*, *HLA‐DPA1*, *TAPBP*, and *HLA‐DRB1* enriched in 30 pathways, including interferon signaling, cytokine signaling in the immune system and PD‐1 signaling pathways; cluster 2 with five genes, *MRPL34*, *IMP3*, *RPS23*, *MRPS18C* and *MRPS34*, which were enriched in 11 pathways, including mitochondrial translation and protein hydroxylation (Table S13B, Supporting Information). The connections between these pathways (e.g., interferon signaling, cytokine signaling, and mitochondrial translation) and cancers have been discussed previously.^[^ ^76^, ^77^, ^78^ ^]^

##### Prioritization of Potential Immune‐Mediated Targets by Evidence Triangulation

Triangulation of causal evidence from genetics, genomics, and clinical trial studies can increase the reliability of the findings and inform novel drug targets that can be prioritized in future clinical trials.^[^ ^32^, ^33^ ^]^ Therefore, we systematically compared 1000 gene‐cancer pairs with robust MR and colocalization evidence using either strong or weak instrumental variables (reported in Table S4A,B, Supporting Information) with target‐indication pairs obtained from PharmaProjects (searched on 25^th^ of March 2023). The 1000 gene‐cancer pairs were combinations of 207 unique genes. Among these genes, 33 (16%; referring to 200 gene‐cancer pairs) were under clinical trial evaluation; 23 of 33 (70%) were anticancer drug targets; 23 of 33 (70%) are still active or complete in clinical trials, with drugs targeting four genes, *LIPA, MAP2K1*, *PSMB5* and *NDUFB3* already having been launched for treatment of cancers or diabetes. Among our findings, nine gene‐cancer pairs from MR overlapped the exact same target‐indication pairs reported in trials (**Table** **2**).

**Table 2:** Genes with robust genetic evidence currently under clinical trial development.

Gene‐cancer MR information			PharamProjects information					
Gene	Entrez Gene ID	Cancer	Drug name	Indications	Indication class	Trail status	Highest Status	Reason for case
***CDC42***	998	Breast cancer	MBQ 167	Breast cancer	Anticancer	Active	Preclinical	/
***PADI4***	23569	Breast cancer	JBI‐1044	Cancer	Anticancer	Active	Phase I	/
***GSTP1***	2950	Prostate cancer	NBF‐006	Lung cancer	Anticancer	Active	Phase I	/
***PIP4K2A***	5305	Prostate cancer	SB‐02829	Cancer	Anticancer	Active	Phase I	/
***HLA‐DRB1***	3123	Cervical cancer	ZYBK‐2	Rheumatoid arthritis	Immunological	Active	Phase I	/
***CDC7***	8317	Breast cancer	Simurosertib	Ovarian cancer, breast cancer, lung cancer, thyroid cancer	Anticancer	Active	Phase II	/
***FADS1***	3992	Lung cancer	dihomo‐gamma‐linolenic acid, DS Biopharma	Cancer	Anticancer	Active	Phase II	/
***CTSS***	1520	Prostate cancer	VGT‐309	Cancer	Anticancer	Active	Phase II	/
***HLA‐DRB5***	3127	Cervical cancer	APOLIZUMAB	lymphoma	Anticancer	Active	Phase II	/
***CHRNA5***	1138	Lung cancer	POZANICLINE	Alzheimer disease	Neurology	Active	Phase II	/
***XPNPEP3***	63929	Prostate cancer	TOSEDOSTAT	Acute myeloid leukemia	Anticancer	Active	Phase II	/
***IL12RB1***	3594	Prostate cancer	JCAR‐020	Ovarian cancer, breast cancer, thyroid cancer, prostate cancer, cervical cancer	Anticancer	Active	Phase III	/
***MDM4***	4194	Prostate cancer	BI‐907828	Ovarian cancer, breast cancer, lung cancer, prostate cancer	Anticancer	Active	Phase III	/
***HLA‐A***	3105	Prostate cancer	Tedopi	Ovarian cancer, breast cancer, lung cancer, prostate cancer	Anticancer	Active	Phase III	/
***PRKAG1***	5571	Prostate cancer	ACADESINE	cardiovascular disease	Cardiometabolic	Active	Phase III	/
***PSMA4***	5685	Lung cancer	MARIZOMIB	glioblastoma multiforme	Anticancer	Active	Phase III	/
***CCNA2***	890	Breast cancer	NEXI‐001	Lung cancer	Anticancer	Active	Phase III	/
***TYMS***	7298	Thyroid cancer	RALTITREXED	neoplasm	Anticancer	Active	Phase IV	/
***PSMB5***	5693	Prostate cancer	Bortezomib	multiple myeloma	Anticancer	Active	Phase IV	/
***RPS23***	6228	Breast cancer	ATALUREN	cystic fibrosis	Lung disease	Active	Phase IV	/
***LIPA***	3988	Breast cancer	sebelipase alfa	Ovarian cancer, breast cancer	Anticancer	Complete	Launched	/
***MAP2K1***	5604	Breast cancer	binimetinib; cobimetinib; trametinib	Ovarian cancer, breast cancer, lung cancer, thyroid cancer	Anticancer	Complete	Launched	/
		Prostate cancer						/
***NDUFB3***	4709	Breast cancer	METFORMIN	Diabetes	Cardiometabolic	Complete	Launched	/
***HLA‐DQA2***	3117	Prostate cancer	MHC‐linked autoimmune disease therapy, PTImmune	Coeliac disease; type 1 diabetes	Immunological	Ceased	Preclinical	Unknown
		Cervical cancer						
***CALR***	811	Breast cancer	Vasostatin	Cancer	Anticancer	Ceased	Preclinical	Unknown
***SLC11A2***	4891	Prostate cancer	XEN‐602	Haemochromatosis	Alimentary/	Ceased	Preclinical	Unknown
					Metabolic			
***CD151***	977	Breast cancer	F‐50065	Cancer	Anticancer	Ceased	Preclinical	Unknown
***ASL***	435	Prostate cancer	PRX‐ASL	Arginosuccinate lyase deficiency	Alimentary/	Ceased	Preclinical	Unknown
					Metabolic			
***TSG101***	7251	Breast cancer	FGI‐102	Hepatitis‐B virus, hepatitis‐C virus, HIV/AIDS, influenza virus	Anti‐infective	Ceased	Phase I	Unknown
***EIF5A***	1984	Breast cancer	SNS‐01	Cancer	Anticancer	Ceased	Phase II	Unknown
***ADAM15***	8751	Breast cancer	AMEP, BioAlliance	Breast cancer, prostate cancer	Anticancer	Ceased	Phase II	Low enrolment rate.
***ADH5***	128	Breast cancer	Cavosonstat	Cystic fibrosis	Lung disease	Ceased	Phase II	Lack of efficacy
***ADNP***	23 394	Prostate cancer	Davunetide	Alzheimer's disease, Progressive supranuclear palsy	Neurological	Ceased	Phase III	Lack of efficacy

**Figure 7:**
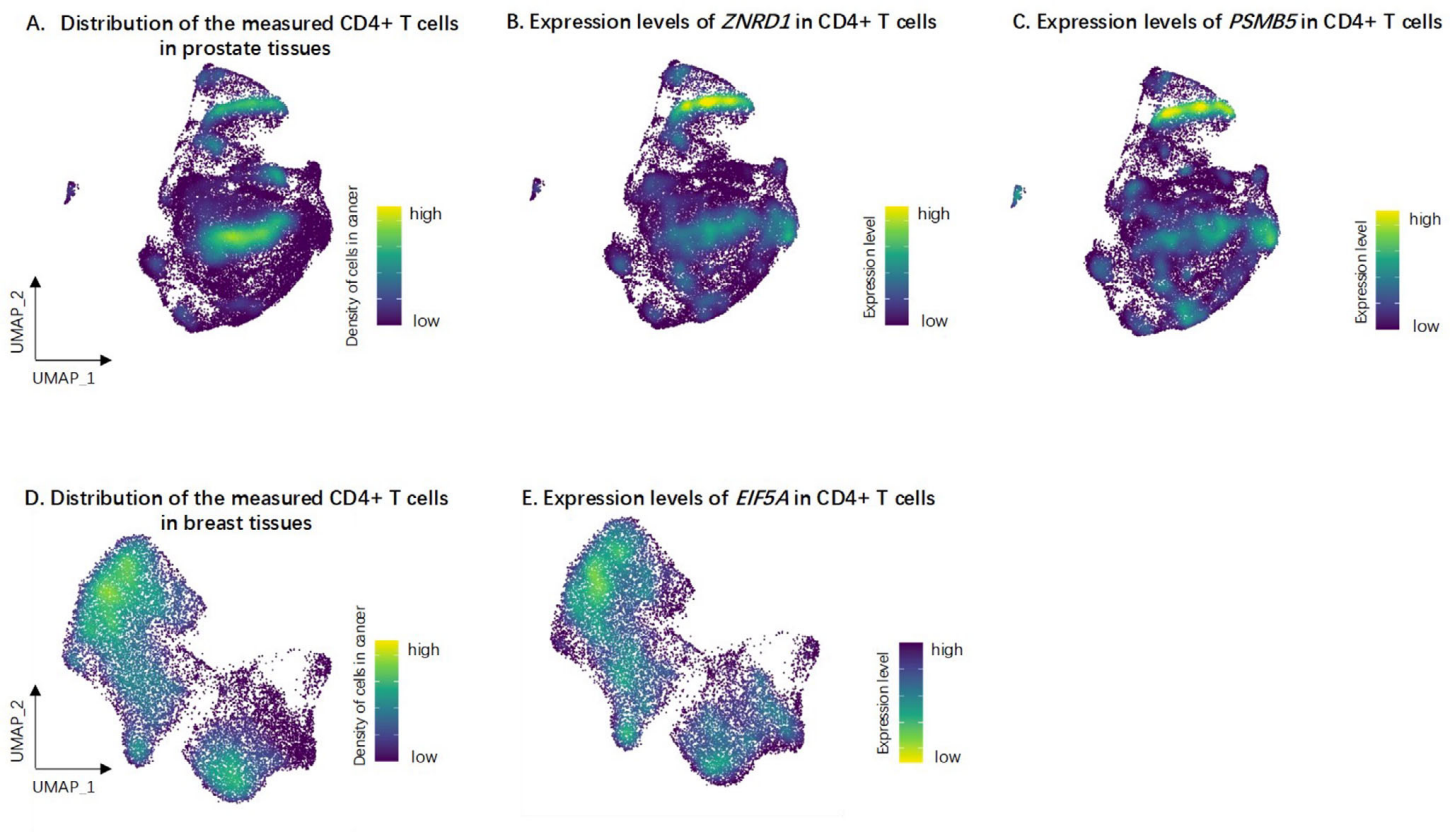
Summary of the scoring system of prioritising most reliable drug target genes for cancer prevention. Abbreviations: DEG, differentially expressed gene; MR, Mendelian randomization.

We further performed triangulation of genetic, biological, and clinical trial evidence for the 1000 top MR estimates and built up a score system to prioritize top signals. The score system included six components: external validation by DEG (+1), MR‐DEG (+1), T‐cell specific effect (+0.5), time point specific effect (+0.5), core genes identified by gene‐set enrichment analysis (+1), and clinical trial evidence of the relevant drug targets (+1). This scoring system prioritized eight genes with multiple layers of evidence (score ≥ 4 out of the total score of 5), which included *EIF5A*, *ZNRD1*, *TYMS*, *RPS23*, *HLA‐A*, *CDC42, HLA‐DRB5*, *HLA‐DQA2* (**Figure** **7**).

*EIF5A* is a positive regulator of the intrinsic apoptotic signalling pathway by p53 class mediator, and tumor necrosis factor‐mediated signalling pathway. The MR analysis showed evidence that genetically‐predicted lower expression of *EIF5A* expression in various CD4+ T cell subtypes was associated with increased risk of breast cancer (OR = 0.96, *p* = 1.7 × 10^−3^). The MR‐DEG test suggested that the *EIF5A* effect is likely to be causal. The DEG analysis showed that the *EIF5A* expression in CD4+ T cell was significantly downregulated in breast tumor cells versus normal cells (logFC = −0.21, *p* = 1.4 × 10^−4^), which is the same direction of effect as our MR findings. The cell‐type specificity analysis further showed that *EIF5A* expression was T‐cell enriched. Importantly, *EIF5A* is the target for an anticancer drug SNS‐01 that was under clinical investigation, although the drug was ceased in Phase II clinical trial. The T‐cell specific effect of *EIF5A* that has been identified in this study may open a new opportunity for this target.

*ZNRD1*, also known as *POLR1H* contains two potential zinc‐binding motifs and may play a role in regulation of cell proliferation. The encoded protein may be involved in cancer progression. Our MR analysis showed that genetically‐predicted increased expression of *ZNRD1* in CD4 + T cells was associated with increased risk of prostate cancer (OR = 1.06, *p* = 1.4 × 10^−3^), and the MR‐DEG test suggested the *ZNRD1* effect as likely causal. The DEG analysis further showed that the expression levels of *ZNRD1* in CD4+ T cells were upregulated in prostate tumor tissues versus normal tissues (logFC = 0.51, *p* = 1.1 × 10^−27^). The cell‐type specificity analysis showed that *ZNRD1* expression was T‐cell enriched.

**Figure 8:**
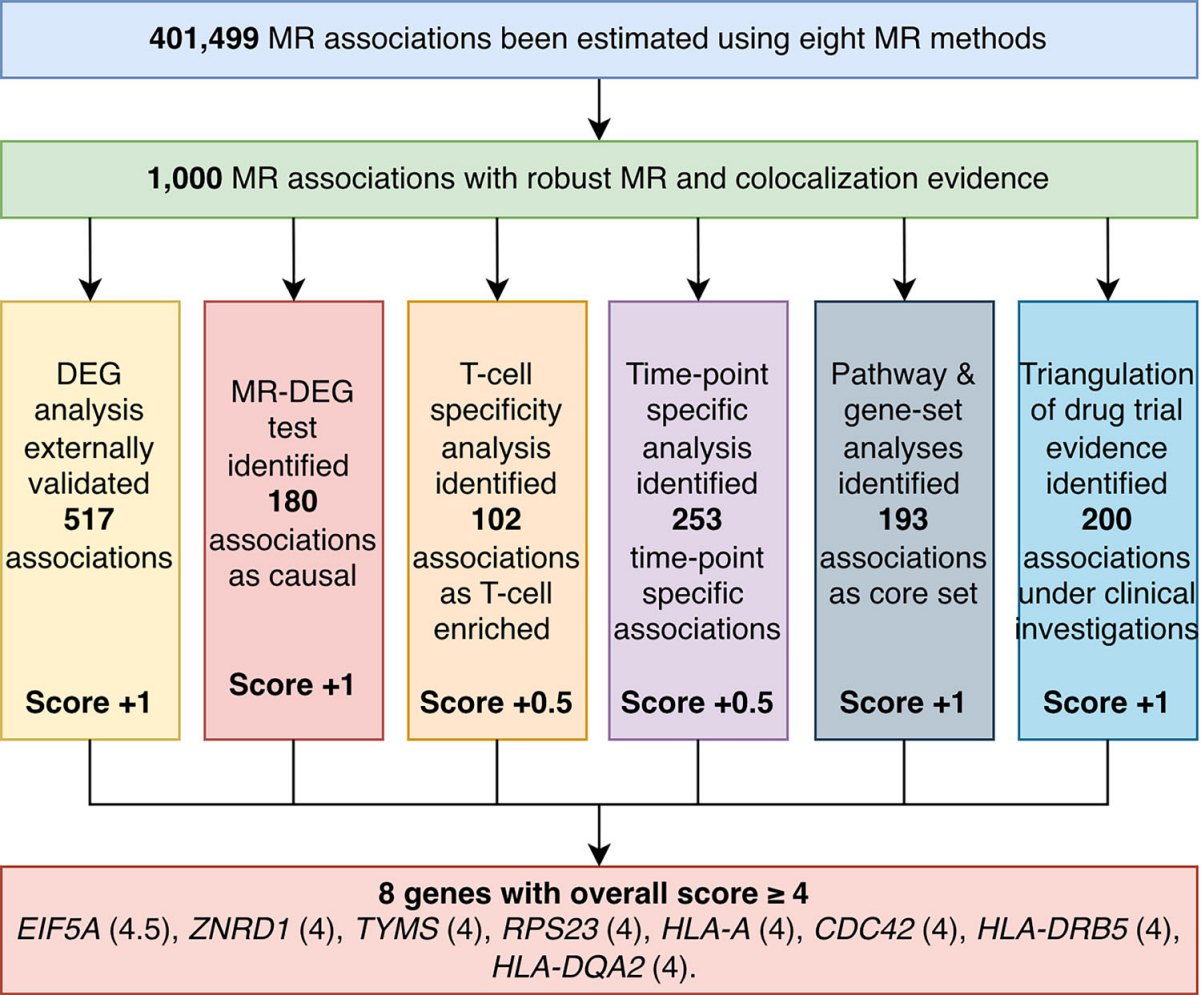
Differentially expressed gene results of three prioritised genes associated with cancers. [Upper] Colocalization between CD4+ T cell distribution in tumour versus normal tissues in prostate cancer patients and expression levels of two genes in CD4+ T cells: A) distribution of the measured CD4+ T cells, light colour refers to density of CD4+ T cells isolated from tumour tissues; B) gene expression levels of *ZNRD1* in CD4+ T cells, light colour refers to high expression levels of the gene; C) gene expression levels of *PSMB5* in CD4+ T cells. [Bottom] Colocalization between CD4 + T cell distribution in tumour versus normal tissues from breast cancer patients and gene expression levels of two genes in CD4+ T cells: D) distribution of the measured CD4+ T cells; E) gene expression levels of *EIF5A* in CD4+ T cells.

For the prioritized genes *EIF5A* and *ZNRD1*, we further conducted an analysis to understand the influence of their expression on downstream genes in cancer cells. We conducted the differentially expressed gene analysis using single‐cell RNA‐seq data in cancer tissues versus normal tissues from Azizi et al. (N = 8 breast tumor vs 8 normal tissues) and Hirz et al. (N = 19 prostate cancer tissues vs 20 normal tissues), followed by pathway enrichment analysis. This analysis showed that comparison of the top 25% with the bottom 25% of individuals (which mimicked those with extremely high or low expression levels of a target gene) revealed a downregulation of expression levels of *EIF5A* in breast cancer and upregulation of expression levels of *ZNRD1* and *PSMB5* on prostate cancer (**Figure** **8**). The top 100 genes influenced by extreme expression levels of *EIF5A* were enriched in 19 pathways (GO enrichment, adjusted *p* of the pathway <0.05), such as actin binding (Table S14A, Supporting Information); the top 100 genes influenced by extreme expression levels of *ZNRD1* were enriched in 28 pathways, including immune‐related pathways such as MHC protein complex binding and MHC class I protein binding (Table S14B, Supporting Information); where the top 100 genes influenced by extreme expression levels of *PSMB5* were enriched in 31 pathways, including MHC class II protein complex binding and MHC protein complex binding (Table S14C, Supporting Information).

It is important to note that our drug target prioritization was mainly based on the use of a *p*‐value threshold for MR evidence (FDR<0.05 based on MR estimates), which we simply use as a heuristic for highlighting estimated effects worthy of follow‐up. Interrogation of our results can incorporate more (or less) stringent thresholds by filtering the gene‐disease effects downloadable from our web browser (https://omicsharbour.org/sc‐eqtl‐mr). As an example of lowering the threshold, additional analyses of existing immune‐mediated drug targets (listed in Table S14C, Supporting Information) validated the effects of *CTLA4* expression on lung cancer and of *METTL3* expression on prostate cancer (Figure S6, Supporting Information).

## 3 Discussion

In this study, we utilized recent advances in single‐cell eQTLs and comprehensively estimated the effects of gene expression dynamics during CD4+ T cell activation on common cancers. By estimating 48 630 effects of gene expression on risks of six types of cancers, we identified 1000 gene‐cancer effects supported by eight MR and colocalization methods (https://www.omicsharbour.com/sc‐eqtl‐mr). 517 of these pairs, refering to 205 unique genes that were differentially expressed using single‐cell RNA‐seq data in cancer tissues. By applying the MR‐DEG tool to the MR signals with potential pleiotropy, 35% of them were defined as likely causal. The cell‐type enrichment analysis showed that 12% of the identified genes showed T‐cell enrichment. 63% of the cancer‐linked genes were only identifiable after activation of T cells, with 64 unique pairs showing distinct causal patterns across activation time points. Integrating with clinical trial evidence, 33 unique cancer‐linked genes were drug targets under clinical trial development, with *LIPA* and *MAP2K1* being targets for already launched anticancer drugs. Collectively, our study emphasizes the value of integrating various dynamic eQTL (termiologies listed in Table 1) MR methods with differentially expressed gene analyses and clinical trial information in identifying novel immune‐related genes, minimizing pleiotropy, and prioritizing drug targets for cancers, such as *EIF5A, ZNRD1*, and *PSMB5*.

Population genetics tools have shown their value in prioritizing causal genes for complex diseases,^[^ ^79^, ^80^, ^81^ ^]^ including cost‐effectiveness, much larger‐scale data, greater resolution of disease end‐points, and providing direct evidence for humans. Previous transcriptome‐wide MR studies have identified gene‐cancer effects using bulk tissues for cells in steady state. For example, Prince et al identified a set of 29 genes associated with breast, prostate, or ovarian cancer using immune cell eQTLs as a validation set.^[^ ^82^ ^]^ Our study validated a few key findings of this study. However, bulk tissue eQTLs may have masked cell‐type‐specific and activation time‐specific effects, thus limiting the ability to prioritize immune‐related targets for cancer prevention.^[^ ^83^ ^]^ Our study integrated population genetics and transcriptomics tools in the context of single‐cell expression data and identified 1000 gene‐cancer effects, of which 30% were not identifiable using other eQTL datasets. This showcases the value of dynamic single‐cell eQTLs in identifying novel causal genes for complex diseases.

Inspired by the idea of evidence triangulation,^[^ ^32^, ^33^ ^]^ we systematically integrated MR and DEG evidence. Despite the different study designs there are two additional points worth mentioning when comparing the two methods. First, we assumed that gene expression of T cells in blood are relatively similar compared with gene expression of T cells in solid tumor cells, both proxied by relevant eQTLs. Previous studies show that the differences in gene expression levels across different tissues are normally quite dramatic, but eQTL signals are relatively stable across tissues.^[^ ^84^, ^85^ ^]^ Our validation CD4+ T cell expression eQTLs in normal tissue and in tumor cells both showed relatively high concordance between blood and tissue samples, which implies that we can consider using eQTLs in blood to proxy eQTLs in tumor cells to some extent. Second, the eQTL MR estimates are qualitatively and directionally comparable with the DEG estimates. We have shown that approximately half of the genes prioritized using T cell eQTL MR showed some DEG evidence to support their associations in T cells involved in tumor tissues. Third, both methods aim to understand the relationships between gene expression and disease, and, by leveraging single‐cell technologies, can now be applied to capture associations at the cell‐type level rather than bulk tissue averages. These results suggest that using eQTLs derived from T cells in whole blood to proxy T cell expression for cancer risk could be a valid assumption. Integrating evidence from both methods, we proposed the concept of MR‐DEG. Similar to Steiger filtering, we positioned MR‐DEG as an easy‐to‐use add‐on test in the QTL MR analysis pipeline to reduce the impact of potential pleitropy. We found that 35% of the irremovable pleiotropic effects have been attenuated after applying this add‐on test. This highlights the value of evidence triangulation, for both external validation and pleiotropy minimization of the top MR findings.

Recent studies have suggested that many immune cell eQTLs, including eQTLs from CD4+ T cells, showed heterogeneous effects across cell types, but the influence of such heterogeneous effects on cancers was unclear. We utilized dynamic single‐cell sequencing data with external stimulation,^[^ ^21^, ^22^, ^23^ ^]^ which allowed us to identify genes with cell‐type and activation‐time specific effects. By comprehensively comparing with four additional eQTL datasets, we quantified that 30% of the gene‐cancer pairs were only detectable using single‐cell dynamic eQTLs. We further quantified that 12% of cancer‐linked genes were T‐cell enriched, among which *EIF5A* and *PSMB5* are targets of anticancer drugs under clinical trial investigation. Five cell types of CD4+ T cells with unclear enrichment beforehand, including TEMRA and nTreg, showed enrichment in the number of causal genes after controlling for the influence of the number of eGenes. This finding aligns with existing evidence that nTreg is known to have anti‐inflammatory functions, supporting anticancer immunity.^[^ ^86^ ^]^ The potential of single‐cell eQTLs in identifying novel risk genes, as well as the immune‐mediated potential of the enriched cell‐types on cancers merits further investigation.

The associations of genetic variants with gene expression of immune cells, including B cells and T cells, have been studied, which suggested that over 35% of the eQTLs were dynamically regulated during cell activation.^[^ ^21^, ^23^ ^]^ These studies open up a unique oppotunity to study the time‐dependent effects of immune cell gene expression on complex diseases. A recent study has modelled time‐dependent cumulative effect of gene expression using principal componenet method.^[^ ^87^ ^]^ Our study demonstrated that 63% of the cancer‐linked genes were only detectable after T cell activation, rather than in resting cells. Furthermore, we observed a set of gene‐cancer pairs with statisticallydifferent MR effects across activation time points. This means even if some of the genes can be identified in resting cells, such temporal patterns will provide value for drugs' biological mechanism of these immune‐mediated genes.

Recent studies showed that the overall failure rate in drug development was over 96%,^[^ ^88^, ^89^, ^90^ ^]^ with lowest likelihood of approval for oncology related drugs.^[^ ^91^ ^]^ Previously, MR has been applied to identify drug targets for complex diseases.^[^ ^13^ ^]^ In this study, we have integrated MR and clinical trial evidence, which prioritized 33 targets that are under current clinical development. Among them, *EIF5A* and *PSMB5*, are targets of anticancer drugs under clinical trial investigation. Despite all the assumptions of this study, we showed that integrating human genetics tools with dynamic single‐cell data and biological information could be a useful approach in scanning immune‐related drug targets for cancers.

Our study also provides a set of methodological insights regarding getting reliable MR estimates using single‐cell QTL data. Previously, we developed omics MR analysis pipelines to prioritize drug targets,^[^ ^13^, ^92^ ^]^ but these pipelines only consider the standalone effect of a single target on a disease despite the fact that genes and proteins interact with each other. In this study, we summarized three major limitations for the single‐cell eQTL data, including limited sample size, weak instrument strength and complex pleiotropy feature (e.g. eQTLs for a specific gene in different cell types). We propose a pipeline to overcome these limitations, which includes selection of valid weak instruments, prioritization of a few methods such as MR‐Robust, debiased IVW and weighted median to get reliable MR estimates using single‐cell eQTL data, and advance the MR‐DEG method. Using this pipeline, we quantified that 35% of the unique gene‐cancer effects with MR and DEG evidence are likely to be causal after minimizing the impact of pleiotropy using MR‐DEG. Further studies are needed to develop MR methods to increase power while accounting for pleiotropy, which can be used as an essential screening of key genes that may have the best potential to be considered as drug targets.

Our study has several limitations. First, population genetics models (including GWAS, transcriptome‐wide association study, MR and colocalization) can provide consistent evidence for the direction of an effect of genetic variants on cancer. Therefore, the different MR estimates across activation time were observed under the assumption that there are interactions between gene expression and activation time for the tested genes. Such interaction causes expression level change across different activation time points, yields different conditionally independent eQTLs of the same gene at different time points, influences the variances explained by eQTLs and finally influences cancer risk differently. The assumption of interaction had been tested by Soskic et al., who found that 34% of the eGenes identified in their study showed dynamic eQTL effects with different activation time points.^[^ ^21^ ^]^ Therefore, the dynamic effects we observed were likely to be plausible. Second, the effects we identified between genes and cancer risks are a key step for target prioritization, but some key features for these targets are still unclear, such as how tumor‐immune microenvironment influences the efficacy of drug targets,^[^ ^20^ ^]^ and the extent to which tumours may develop resistance against these therapeutic targets.^[^ ^93^ ^]^ Future preclinical and clinical investigations are warranted for these targets to better understand these features. Third, given different statistical power and potential winner's curse in the eQTL datasets, the instrument strength of the eQTLs, indexed by F‐statistics, ranged from 10 to 1538. Therefore, we did not expect the same level of evidence for the dynamic and non‐dynamic eQTL MR analyses. There is a possibility that some MR signals showed similar effects in both analyses, but one with a wider confidence interval compared with the other. Fourth, when considering tissue‐specificity of the eQTLs, four of the five data sources identified eQTLs in blood samples, but tissue‐specific eQTLs from GTEX were also included. Although eQTLs were relatively stable across tissues, we still need to acknowledge that tissue‐specific effects of the eQTLs may create complexity when interpreting the results. Fifth, the resolution of the RNA‐seq data from these studies was also quite different. Given the limitation of the single‐cell RNA‐seq technology, the sequencing depth of single‐cell RNA‐seq is lower compared to bulk RNA‐seq. Further single‐cell eQTL studies are needed with more participants, more cells and better RNA‐seq depth. Sixth, since the outcome data we used were from case‐control GWAS studies, the gene‐cancer pairs that we identified are potential immune‐related targets for cancer prevention rather than treatment. Further studies are needed to estimate the effects of immune cell expression on cancer progression using specific methods, such as the Slope‐Hunter approach,^[^ ^94^, ^95^ ^]^ which will directly inform drug targets for cancer treatment. Cancer survival in a case‐only setting will be another attractive angle to be investigated in future studies.^[^ ^96^ ^]^ Seventh, we identified a set of cancer‐linked genes in the MHC region. The region is highly related to immunity, but genetic variants within this region have a very complex LD structure, and therefore we could not make strong claims of causality for genes within this region, although an attempt has been made to develop methods for genetic causal inference in such regions.^[^ ^97^ ^]^ Finally, given that we only have single‐cell eQTL data from European ancestry groups, we need data from other ancestries to test the generalizability of ancestry‐specific effects on cancers.

In summary, this study prioritized immune‐mediated targets for cancers using population genetic tools. The cell‐type‐ and activation time‐specific effects of gene expression on cancer that have been illustrated here provide key evidence to support the design of future drugs targeting prioritized genes in specific cell‐type and activation time points. Our findings and the analysis pipeline for single‐cell eQTL MR will support future study design, analysis, and interpretation of dynamic and single‐cell transcriptome MR.

## 4 Experimental Section

### Data Sources

Dynamic CD4+ T cell expression quantitative trait loci (eQTL) data were accessed from Soskic et al.^[^ ^21^ ^]^ The additional eQTL datasets were accessed from the OneK1K, DICE, GTEX, and eQTLGen^[^ ^12^, ^22^, ^23^, ^34^ ^]^ datasets (Table S1, Supporting Information).

The expression data were measured using RNA‐seq in bulk tissues or single‐cell RNA‐seq in immune cells. For Soskic et al, 119 European ancestry individuals with single‐cell sequencing data in CD4+ T cells were selected as samples for the main analysis, and up to 31 684 Europeans with expression data from OneK1K, DICE, eQTLGen, and GTEX studies were selected as comparison samples.

For the disease outcome data, GWAS data were assembled from up to 1 120 977 European samples (with up to 122 977 cancer cases) with genetic association information from the cancer GWAS data implemented in the IEU OpenGWAS database^[^ ^38^, ^39^, ^40^, ^41^, ^42^ ^]^ and Global Biobank Meta‐analysis Initiative^[^ ^43^ ^]^ (GBMI; Table S2, Supporting Information). Detailed information about these cohorts is listed below.

### Soskic et al

Soskic et al. mapped dynamic eQTL (termiologies listed in Table 1) effects of CD4+ T cells during cell activation using single‐cell transcriptomics technology.^[^ ^21^ ^]^ This study isolated and stimulated naive and memory CD4^+^ T cells in the resting state (0 h), before dividing (16 h), after the first cell division (40 h), and after acquiring effector functions (5 d) from 119 healthy individuals and performed single‐cell RNA sequencing. 655 349 CD4^+^ good quality T cells were extracted and further stratified into 38 cell clusters based on their correlated patterns of gene expression. The gene expression data were linked with genetic variants using a linear regression model, which provided dynamic eQTL data for CD4^+^ T cells.

### OneK1K

The OneK1K cohort consists of 982 healthy Northern Europeans with no active infection reported at the time of sample collection. In this cohort, single‐cell RNA sequencing data from 1 267 758 peripheral blood mononuclear cells were acquired. The single‐cell eQTL study using these data developed a framework to independently classify each cell into one of 14 different immune cell types across the myeloid and lymphoid lineages based on their transcriptional profiles.^[^ ^23^ ^]^ The immune cell types included B cells, T cells, nature killers, monocytes, and dendritic cells. The single‐cell eQTL were identified for each of the 14 immune cell types.

### DICE

The DICE (Database of Immune Cell Expression, Expression quantitative trait loci (eQTLs) and Epigenomics) project was established in 2014 and aims to understand the role of common genetic variations in human disease as well as the cell‐type specific effects on immune cell gene expression.^[^ ^22^ ^]^ The dataset was designed to reveal the effects of disease risk‐associated genetic polymorphisms on specific immune cell types.

### GTEX

The Genotype‐Tissue Expression (GTEx) project was launched in 2010 with the aim of characterizing genetic effects on gene expression across a large number of human tissues to link the molecular mechanisms of genetic associations with complex diseases and traits. The GTEx v8 dataset consists of 838 donors and 17 382 samples from 52 tissues and 2 cell lines, with adequate power to detect eQTL in 49 tissues.^[^ ^34^ ^]^

### eQTLGen

The eQTLGen Consortium was set up to investigate the genetic architecture of blood gene expression and to understand the genetic underpinnings of complex traits, which consists of 31 684 blood and PBMC samples from 37 datasets.^[^ ^12^ ^]^ In total, 25 482 (80%) of the samples were whole‐blood and 6202 (20%) were PBMC samples. The majority of samples were from European ancestry. The eQTLGen Consortium performed a large‐scale meta‐analysis and identified cis‐eQTL for 16 987 genes, trans‐eQTL for 6298 genes, and eQTS effects for 2568 genes. The bulk eQTLGen project is currently in its second phase, which is focused on a large‐scale genome‐wide meta‐analysis in blood.

### IEU Open GWAS Database

The IEU GWAS database^[^ ^39^ ^]^ underpins the IEUs flagship MR‐Base analytical platform (www.mrbase.org) and was used to identify associations between genetic variants and diseases. This database is hosted by the MRC Integrative Epidemiology Unit (MRC IEU) at the University of Bristol and contains over 250 billion genetic association records from more than 40 000 human traits.

### Global Biobank Meta‐Analysis Initiative (GBMI)

Global Biobank Meta‐analysis Initiative (GBMI) is a collaborative network containing multiple biobanks collaborating through meta‐analysis with established resources of genotype, phenotype, and GWAS to develop a global and growing resource for human genetics research (https://www.globalbiobankmeta.org/). GBMI currently represents 2.6 million research participants with health and genetic data from 21 biobanks across four continents. It incorporates diverse ancestries in genetic studies by including biobank samples from 6 main populations and 14 endpoints selected based on the common interest of the contributing biobanks.^[^ ^98^ ^]^ Incorporating samples with diverse ancestries in the biobank meta‐analysis enables comparison of effect sizes of genomic loci across ancestry. Also, the sex‐stratified meta‐analysis allows for comparing effect sizes of the genomic loci between sexes.

### Statistical Analyses–Genetic Instrument Selection of Dynamic and Non‐Dynamic eQTLs During CD4+ T Cell Activation

In this study, the genetic variants associated with dynamic eQTLs were used as genetic instruments for the MR analysis. The instrument selection process was started by accessing the eQTL data from five studies. All conditionally independent eQTLs that were associated with gene expression were selected, with pair‐wise LD r^2 ^< 0.001 to avoid the issue of collinearity in the MR model. To fit with the data requirements of the MR and colocalization analyses, eQTLs with full summary statistics available in the *cis*‐acting regions were only selected. For the main MR analysis, 11 021 dynamic eQTLs of 1817 genes in 119 Europeans from Soskic et al (Table S1A, Supporting Information) were selected.

For the non‐dynamic eQTL MR analysis, the following eQTLs were selected as instruments (Figure 1): 1) 291 conditionally independent eQTLs of 284 genes expressed in three types of CD4+ T cells derived from single‐cell sequencing data of 982 Europeans from the OneK1K cohort^[^ ^23^ ^]^ (Table S1C, Supporting Information); 2) 787 immune cell eQTLs of 82 genes derived from the DICE consortium^[^ ^22^ ^]^ (Table S1D, Supporting Information); 3) 5279 tissue‐specific eQTLs of 177 genes from GTEX v8^[^ ^34^ ^]^ (Table S1E, Supporting Information); and 4) 162 whole blood eQTLs of 160 genes from the eQTLGen consortium^[^ ^12^ ^]^ (Table S1F, Supporting Information). In total, 6519 instruments for 392 genes (5238 exposures) were selected to compare with the top findings identified from the main analysis.

### Genetic Instrument Validation

An instrument selection process was applied to select instruments that fit better with the MR assumptions, which included the following three validation steps.

### Validation of Instrument Strength

To quantify the statistical power of the eQTLs, the strength of the genetic predictors of each tested variant was estimated using F‐statistics. If any eQTLs had F‐statistics lower than the widely used threshold of 10, they were considered to have limited power (potentially causing weak instrument bias^[^ ^99^, ^100^ ^]^) and were removed from the MR and follow‐up analyses.

### Validation of Instruments Using Directionality Test

From a drug development point of view, a valid drug will influence the expression level of a gene, altering disease risk subsequently. Therefore, a directionality test was conducted to better understand the direction of effect of the MR findings. Steiger filtering was used^[^ ^101^ ^]^ to test the directionality of the gene expression–disease associations for all candidate instruments. Any eQTLs with Steiger filter flag as FALSE (which means the eQTL explains more of the variance in the outcome than it does the variance in the exposure) were removed from the MR and follow‐up analyses (Table S1A–E, Supporting Information) to avoid potential for reverse causality^[^ ^102^ ^]^ (where genetic predisposition to cancer has an effect on the gene).

### Validation of cis‐eQTLs as Instruments

Only *cis*‐acting eQTLs within 500 KB of the transcripts/genes were selected as genetic instruments, since *cis*‐acting eQTLs are more likely than *trans*‐acting eQTLs to have gene‐specific effects.^[^ ^103^ ^]^ For genes with multiple instruments, LD clumping was applied to identify top and secondary independent eQTLs.

### Re‐Selection of Weaker Genetic Instruments of Dynamic eQTLs During CD4+ T Cell Activation

Given the sequencing depth of single‐cell RNA‐seq, one strong instrument per exposure (cell‐type and time point‐specific gene expression profiles) was identified, which might limit the ability to identify reliable MR estimates and detect potential pleiotropy. To increase the number of instruments, a pipeline was proposed to identify weaker instruments. In this pipeline, a threshold of genetic association *p*‐value threshold (*p* < 0.05) and F‐statistic (> = 5) to select sufficient instruments for MR sensitivity analyses.Steiger filtering and LD clumping (LD r^2^ < 0.1, referring to the 1000 Genome Project Europeans) was further applied for the selected variants. After selection, 47 051 valid instruments were totally identied for 11 007 exposures, in which 845 of the exposures only had one instrument, and the remaining 10 162 exposures had two or more instruments (Table S1B, Supporting Information). These instruments were used as additional data to validate the MR findings using strong instruments.

### Outcome Selection in the IEU Open GWAS Database and GBMI

Cancer GWASs were selected from the IEU Open GWAS database and GBMI using three criteria:

1. The GWAS summary statistics were available in the two databases.
2. Number of cancer cases over 1000, so that the logistic model used for the GWAS provided good power.
3. The eQTL data that were applied were obtained from both males and females; sex‐combined disease GWAS was used for the main MR analysis, so the exposure and outcome of the MR were equally represented in the population.

Based on these criteria, six common cancers were selected as the outcomes for the MR analysis, including: thyroid cancer, breast cancer, lung cancer, ovarian cancer, prostate cancer, and cervical cancer. In addition, pan‐cancer from UK Biobank + FinnGen was used as a validation outcome. The sample sizes of the six cancer GWASs were from 27 209 to 1 120 977 (Table S2, Supporting Information).

### Main MR Analyses of Dynamic Gene Expression of CD4+ T Cell on Cancers

In the main MR analysis, the effects of dynamic gene expression levels of CD4+ T cell was estimated on the six selected cancer types. To best represent the genetic signals in the cis‐acting region and boost power, one of the analyses was conducted depending on how many eQTL instruments were selected. For gene expression with only one instrument, Wald ratio analysis was conducted^[^ ^44^ ^]^ to estimate the effects of gene expression on cancers. For gene expression with two or more instruments, the conditional independent eQTLs were used as genetic instruments and an inverse variance weighted (IVW) approach was applied. To correct for multiple testing, the MR estimates with FDR corrected *p*‐value < 0.05 were used to select candidate gene‐cancer pairs for follow up analyses (number of tests for MR = 48 630). The MR analyses were conducted using the TwoSampleMR R package (https://github.com/geneinmylife/single‐cell‐Mendelian‐randomization).^[^ ^104^ ^]^

### MR Analyses of Dynamic Gene Expression of CD4+ T Cell on Cancers Using Weaker Instruments

For MR analysis using weaker instruments, 845 exposures can only apply the Wald ratio method since only one instrument was identified for each of them. For the remaining 10 162 exposures, seven additional MR methods were applied, including weighted median,^[^ ^105^ ^]^ weighted mode,^[^ ^106^ ^]^ MR‐Robust,^[^ ^107^ ^]^ cML‐MA,^[^ ^108^ ^]^ MR‐PRESSO^[^ ^109^ ^]^ (need four or more instruments), debiased IVW,^[^ ^110^ ^]^ and MR‐RAPS,^[^ ^111^ ^]^ to validate the main MR findings and make a comparison of the performance of these methods in single‐cell QTL MR analysis. The MR estimates were corrected for multiple testing using FDR‐corrected *p*‐value < 0.05 as the threshold. These analyses were conducted using “TwoSampleMR,” “MendelianRandomization,” “MR‐PRESSO,” and “mr.raps” R packages. MR‐Egger was not applied here since all the instruments we used were within the cis‐acting regions of the tested genes rather than across the whole genome. Therefore, the INSIDE assumption of MR‐Egger is not likely to hold in this case.

### Sensitivity Analysis of Candidate MR Signals

To increase the reliability of the MR signals, we applied a set of sensitivity analyses for the candidate MR signals.

### Genetic Colocalization Analysis of the Candidate MR Signals

Results that passed the FDR *p*‐value threshold of *p* < 0.05 using Wald ratio or IVW approaches were evaluated using genetic colocalization analysis. The purpose of this analysis was to distinguish MR signals from gene‐cancer pairs confounded by LD (see Figure 5A). Two sets of colocalization analyses were applied to obtain more reliable colocalization evidence. First, an approximate colocalization analysis that was developed was applied, which is noted as LD check.^[^ ^13^ ^]^ The LD r^2^ was estimated^[^ ^2^ ^]^ between each eQTL against all variants with GWAS *p* < 1 × 10^−3^ in the cis‐acting region associated with the relevant cancer outcome. R^[^ ^2^ ^]^ of 0.7 between the eQTL and any of the cancer outcome variants was used as evidence for approximate colocalization.

Second, conventional genetic colocalization analysis was applied using the “coloc” R package.^[^ ^25^ ^]^ For the colocalization analyses, the default prior probabilities that a variant is equally associated with each phenotype (p_1_ = 1×10^−4^; p_2_ = 1×10^−4^), were used, and both phenotypes jointly (p_12_ = 1 × 10^−5^). A colocalization probability PP.H4 / (PP.H3+PP.H4) > 70% in this analysis would suggest that the two genetic association signals are likely to colocalize within the test region, as this analysis was based on candidate MR signals, there is some evidence to support the effect of gene expression on diseases already, so the colocalization probability was slightly relaxed to include more candidate drug targets for the follow up analyses.

### Comparison MR Analysis Using Non‐Dynamic eQTL Datasets

In the comparison MR analysis, for any gene‐cancer pairs that passed the FDR threshold p < 0.05, independent eQTLs were selected from four independent eQTL studies: OneK1K with immune cell eQTLs derived from single‐cell sequencing; DICE with immune cell eQTLs from bulk tissues; GTEX with tissue‐specific eQTLs in 49 tissues, and eQTLGen with whole‐blood eQTLs. For consistency, the same instrument selection and validation steps were applied for these additional eQTL datasets. After selection, 291 conditionally independent eQTLs of 284 genes expressed in three types of CD4+ T cell derived from OneK1K cohort (Table S1B, Supporting Information), 787 immune cell eQTLs of 82 genes derived from the DICE consortium (Table S1C, Supporting Information), 5281 tissue‐specific eQTLs of 178 genes from GTEX v8 (Table S1D, Supporting Information), as well as 162 whole blood eQTLs of 160 genes from eQTLGen consortium (Table S1E, Supporting Information) were selected as instruments for the comparison MR analysis. The same analysis pipeline, including F‐statistics check, Steiger filtering, MR, colocalization, and LD check, was applied for the comparison MR analysis. A Benjamin‐Hochberg FDR of 0.05 and colocalization probability PP.H4 > 70% were applied to select MR estimates with additional genetic evidence.

To define novel findings of this study, the gene‐cancer pairs identified in the dynamic eQTL MR analysis were systematically compared with those detected in the non‐dynamic eQTL MR analysis. The Venn diagram was drawn to estimate the proportion of gene‐cancer pairs that were only identifiable using dynamic eQTLs in the main MR analysis.

### Cell‐Type, Cancer‐Type, and Activation Time Specific Analyses–Cell‐Type Specific Analysis

In this analysis, the type of T cells that showed enriched MR signals in cancers was aimed to be estimated. The number of gene‐cancer pairs identified in each of the 246 cell‐types, activation time, and cancer‐type combination was first estimated. Since Soskic et al. reported that different cell‐types showed quite different numbers of eGenes, with more eGenes being identified in naïve CD4+ T cells than memory T cells. The correlation of the number of MR signals with the number of eGenes for each of the cell state/activation time point combinations was therefore estimated using Spearman's correlation. Given the strong correlation between the number of MR signals and the number of eGenes for each combination, the cell‐type enrichment of the MR signals adjusted for the number of eGenes was estimated. Fisher's exact test was applied to test for enrichment between the proportion of eGenes versus MR signals in a certain cell type against the proportion of eGenes versus MR signals in all cell types.

To further understand the enrichment pattern of MR signals in naïve and memory CD4+ T cell, 255 gene‐cancer pairs with robust genetic evidence in either naïve or memory T cells were selected (Table S9, Supporting Information). The proportion of MR signals that showed up in naïve only, memory‐only, or both T cells was estimated. For those pairs with MR evidence in both cell states, the pair‐wise Z score test^[^ ^112^ ^]^ was used to estimate whether the MR estimates of expression in naïve and memory T cells on the same cancer overlapped with each other.

In addition, whether the identified genes showed T‐cell‐specific effects or just a general signal in many different immune cell types was aimed to be estimated. For the 207 unique cancer‐associated genes with MR and colocalization evidence, their immune‐cell specificity was estimated using cell‐type‐specific RNA‐seq data from the DICE consortium.^[^ ^22^ ^]^ The DICE study provided cell‐type‐specific expression of the genes in 15 immune‐cell types. If the expression levels of a gene in T cells are five or more times higher than its expression levels in other immune cell types, this gene is defined as T‐cell‐enriched. In addition, if the expression levels of a gene in T cells are two or more times higher than its expression levels in other immune cell types, this gene is identified as T‐cell enhanced.

### Cancer‐Type Specific Analysis

Two aims were set up in the cancer‐type‐specific analysis. First, it was aimed to estimate whether the expression of CD4+ T cells was enriched in any specific cancer type. Second, it was also aimed to estimate where expression of the same gene may show an effect on multiple cancer types. To achieve the first aim, Fisher's exact test was applied to estimate the proportion of gene‐cancer pairs identified for each cancer type against that for all cancer types, adjusting for the influence of the number of cases of the cancer GWAS data. For the second aim, we extended the 994 top MR signals that passed FDR corrected threshold of 0.05 to all MR records of the same gene‐cell‐type‐time point combination without any *p*‐value cutoff. This resulted in 4844 MR records for the 952 gene‐cell‐type‐time point combinations (Table S10, Supporting Information). Among these records, the percentage of combinations with MR evidence in two or more cancers was estimated, using FDR corrected *p* < 0.05 or MR *p* < 0.05 as a cutoff for defining MR signals.

### Activation Time Specific Analysis

This analysis aimed to achieve a few goals. First, the percentage of cancer‐linked genes (identified by our MR study) that can only be identified in resting T cells was tried to be estimated, and further, the proportion of genes that showed effects on cancers at multiple activation time points was estimated. Second, to systematically investigate the activation time‐dependent effects of CD4+ T cell expression on cancers, it started with all 48 630 gene‐cancer pairs tested in this study and selected 742 MR records of 455 gene‐cancer pairs with robust MR signal (FDR < 0.05) in one activation time point and with MR estimates (can either passed or not passed the FDR threshold) in at least one other time points (Table S11A, Supporting Information). For each of the 455 pairs, the time point with the most distinct MR effect was set as reference. Pair‐wise Z‐score tests were applied between the reference MR effect and effects at each of the other time points. A pair‐wise Z‐score test *p*‐value lower than 0.05 was selected as a threshold of gene‐cancer pairs with distinct effects across activation time points.

### Pathway and Gene‐Set Analyses–Pathway Analysis

The pathway enrichment analyses were performed using the default setting of Metascape,^[^ ^66^ ^]^ which implemented databases such as REACTOME, GO databases. The cancer‐associated genes identified in this MR study were listed as an unordered query and using all genes included in the database as background. Pathways with entities FDR < 0.05 were listed as those with enrichment evidence. Enriched pathways were visualized in R using the ggplot2 package.

### Gene‐Set Enrichment Analysis

The protein–protein interaction (PPI) network of the 186 cancer‐linked genes was identified using StringDB database,^[^ ^72^, ^73^ ^]^ with a minimum required interaction score of 0.4. The core genes for all six cancers and each cancer type were estimated using the Cytoscape (version 3.6.1)^[^ ^74^ ^]^ plugin cytoHubba, respectively.^[^ ^75^ ^]^ Those cancer‐associated genes with maximal clique centrality [MCC] ≥ 9, a parameter to discover featured nodes from the PPI network, have been identified as core genes in the gene‐set enrichment analysis (Table S13, Supporting Information).

### Differentially Expressed Gene Analysis Using T Cell Expression Data in Cancer Tissues

For the 224 unique‐gene cancer pairs with robust MR and colocalization evidence, single‐cell gene expression data of conventional T cells were searched and extracted from various publicly available resources: three breast cancer datasets (under accession codes GSE195665, GSE114725, GSE161529); three cervical cancer datasets (E‐MTAB‐11948, GSE208653, E‐MTAB‐12305); three lung cancer datasets (GSE99254, GSE131907, E‐MTAB‐13526); ovarian cancer data from Xu et al. (GSE184880); three prostate cancer datasets (GSE185334, www.prostatecellatlas.org, GSE181294); and three thyroid cancer datasets (GSE184362, GSE193581, GSE158291).^[^ ^49^, ^50^, ^51^, ^52^, ^53^, ^54^, ^55^, ^56^, ^57^, ^58^, ^59^, ^60^, ^61^, ^62^, ^63^, ^64^ ^]^ All single‐cell RNA‐seq data obtained from public databases were re‐analyzed using the Seurat package v5.0.3.Table^[^ ^113^ ^]^ Low‐quality cells were filtered out based on the following criteria: cells with nFeature_RNA ≤ 200 or percent.mt > 5 were removed. The SCTransform function was then applied to normalize the data.

Differentially expressed gene analyses were conducted using the FindAllMarkers function (settings: min.pct = 0.1, |logfc| threshold = 0.25) to understand the expression patterns of the cancer‐associated genes in T cells in normal tissue versus T cells in tumor tissues. The dataset batch was set as a covariate in the model to adjust for the influence of potential confounding factors. Colors refer to the gene‐weighted kernel density estimated using the Nebulosa R package.^[^ ^114^ ^]^

### Concept and Application of MR‐DEG

To triangulate additional evidence, evidence was combined from DEG and MR analysis. These two methods both aim to understand the association of expression of a gene in one cell‐type with an outcome (e.g., a type of cancer). However, DEG analysis used totally independent data sources and analytical pipelines compared with MR (expression data versus eQTL data; tumor‐normal tissues versus case‐control GWAS of cancer). Instead of simply comparing the MR and DEG results of the same gene on the same cancer site, it is considered that DEG analysis can estimate the gene‐cancer associations of the potential pleiotropy genes as well. Using these DEG results, potential pleiotropic genes that were not removable solely using MR could be excluded. For example, if an eQTL is associated with the expression levels of three genes A, B, and C, then this eQTL is potentially pleiotropic, and the Wald ratio method is not able to show which gene is the causal gene. However, differentially expressed gene analysis could be conducted on all three genes against the cancer outcome. If the DEG analysis showed that expression of Gene B and Gene C was not associated with the cancer outcome, then it can be concluded with more confidence that Gene A is likely to be the causal gene (see Figure S3, Supporting Information). Using this approach, a certain level of pleiotropy will be minimized. Therefore, MR‐DEG method could be used to strengthen MR causal estimates.

MR‐DEG approach was applied to the 252 MR signals with DEG evidence but also showed potential pleiotropy (instruments associated with expression levels of two or more genes with *p* < 1 × 10^−4^, Table S4A, Supporting Information). DEG analysis was conducted using single‐cell RNA‐seq data in CD4+ T cells from 16 studies^[^ ^49^, ^50^, ^51^, ^52^, ^53^, ^54^, ^55^, ^56^, ^57^, ^58^, ^59^, ^60^, ^61^, ^62^, ^63^, ^64^ ^]^ and applying the MR‐DEG method.

### Triangulation of Gene‐Cancer MR Signals with Clinical Trial Evidence

Previous studies suggested that drug targets with genetic evidence were more likely to be successful in clinical trials.^[^ ^79^ ^]^ Here, it is assessed whether drug targets under clinical trials were enriched for gene‐cancer pairs obtained in the present study using population genetics tools. For the 207 cancer‐linked genes identified in this study, their Entrez Gene IDs were mapped using gprofiler (https://biit.cs.ut.ee/gprofiler/convert). This allowed us to match the cancer‐associated genes with corresponding targets in the Pharmaprojects database from Citeline (https://pharmaintelligence.informa.com/; searched on 25^th^ of March 2023), which manually annotated target‐indication pairs (Tablfor drugs under clinical trials. The clinical trial information of the drug targets that can be mapped to the 186 cancer‐linked genes was collected, which includes drug name, indication, indication class, trial status, and highest trial status (Table 2). The percentage of cancer‐associated genes that were under clinical trials was estimated.

### Comparison Between MR Evidence and Existing Immune‐Mediated Drugs

To compare MR evidence of this study with existing immune‐mediated drugs, the literature was comprehensively searched, and a list of 54 genes that are targets of existing immune‐mediated drugs (Table S14A, Supporting Information) was identified. Two genes, *CTLA4* and *METTL3*, obtained valid MR results in this study, which were selected in this analysis. The MR estimates of expression levels of *CTLA4* and *METTL3* across 19 T cell states and five activation time points on six cancers were extracted from the Omics Harbour single‐cell eQTL MR browser (https://www.omicsharbour.com/sc‐eqtl‐mr). The MR estimates with MR *p*‐value < 0.05 were listed in Table S14B (Supporting Information). The forest plot was drawn to compare the effect estimates of *CTLA4* expression across T cell‐types/activation time on cancers.

## Conflict of Interest

T.R.G. and G.D.S. have received research funding from various pharmaceutical companies to support the application of Mendelian randomization to drug target prioritization. G.D.S. reports Scientific Advisory Board membership for Relation Therapeutics, Insitro and Bristol Myers Squibb. T.R.G. receives funding from Biogen, Roche and GSK for unrelated research.

## Author Contributions

J.Z., Q.Y., H.L., H.Z., and S.W. contributed equally to this work. J.Z., T.R.G., J.L., and Y.B. designed the study, wrote the research plan, and interpreted the results. J.Z., Q.Y., X.W., and H.Z. have accessed and verified the underlying data. J.Z. and X.W. undertook the main MR and sensitivity MR analyses. H.Z. conducted the follow‐up MR and wrote relevant sections. H.L. conducted the differentially expressed gene analysis. Y.L. and T.R.G. built up the web app. Y.Y., X.H., L.Y., and H.Y. supported relevant analyses. J.Z. wrote the first draft of the manuscript with critical comments and revisions from all other authors. J.Z. is the guarantor. The corresponding author attests that all listed authors meet authorship criteria and that no others meeting the criteria have been omitted. All authors read and approved the final version of the manuscript.

## Supporting information



Supplemental Figure 1‐6

Supplemental Table 1‐14

## Data Availability

The Mendelian randomization and follow‐up analyses scripts are available via GitHub (https://github.com/geneinmylife/single‐cell‐Mendelian‐randomization). The GWAS summary data used in this study are publicly available via the IEU OpenGWAS database (https://opengwas.io). The Mendelian randomization estimates estimated in this study can be accessed via the single cell eQTL MR browser of Omics Harbour platform (https://www.omicsharbour.com/sc‐eqtl‐mr). Further information and requests for resources should be directed to and will be fulfilled by the lead contact, Jie Zheng (zj12477@rjh.com.cn).
