# Supplementary material for: Integrating Single‐Cell Transcriptome‐Wide Mendelian Randomization and Differentially Expressed Gene Analyses to Prioritize Dynamic Immune‐Related Drug Targets for Cancers
Source: Adv Sci (Weinh). 2025 Nov 11;12(46):e07451. doi: 10.1002/advs.202507451 (PMC12697907; doi:10.1002/advs.202507451)
Supplement: Supplementary file 1 — Supplemental Figure 1‐6 [file ADVS-12-e07451-s001.docx]

## Supplementary Figures

**
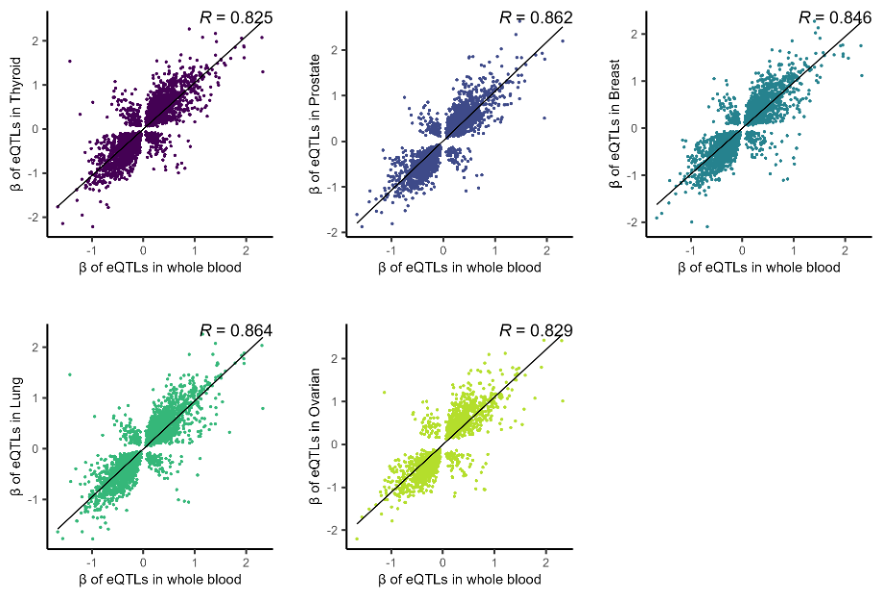
**

**Figure S1**. Correlation between eQTLs in whole blood vs in cancer stie tissues.


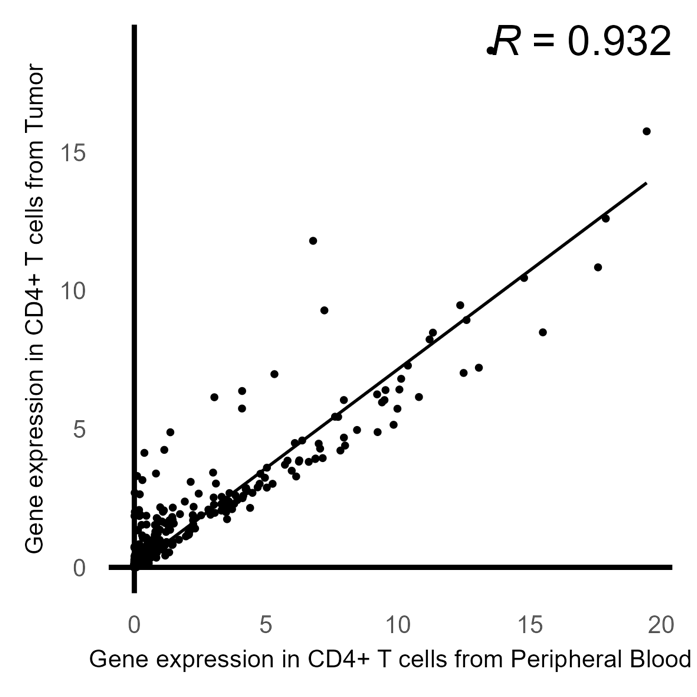


**Figure S2**. Correlation between gene expression in CD4+ T cell from peripheral blood vs that in CD4+ T cell from Tumor tissues.


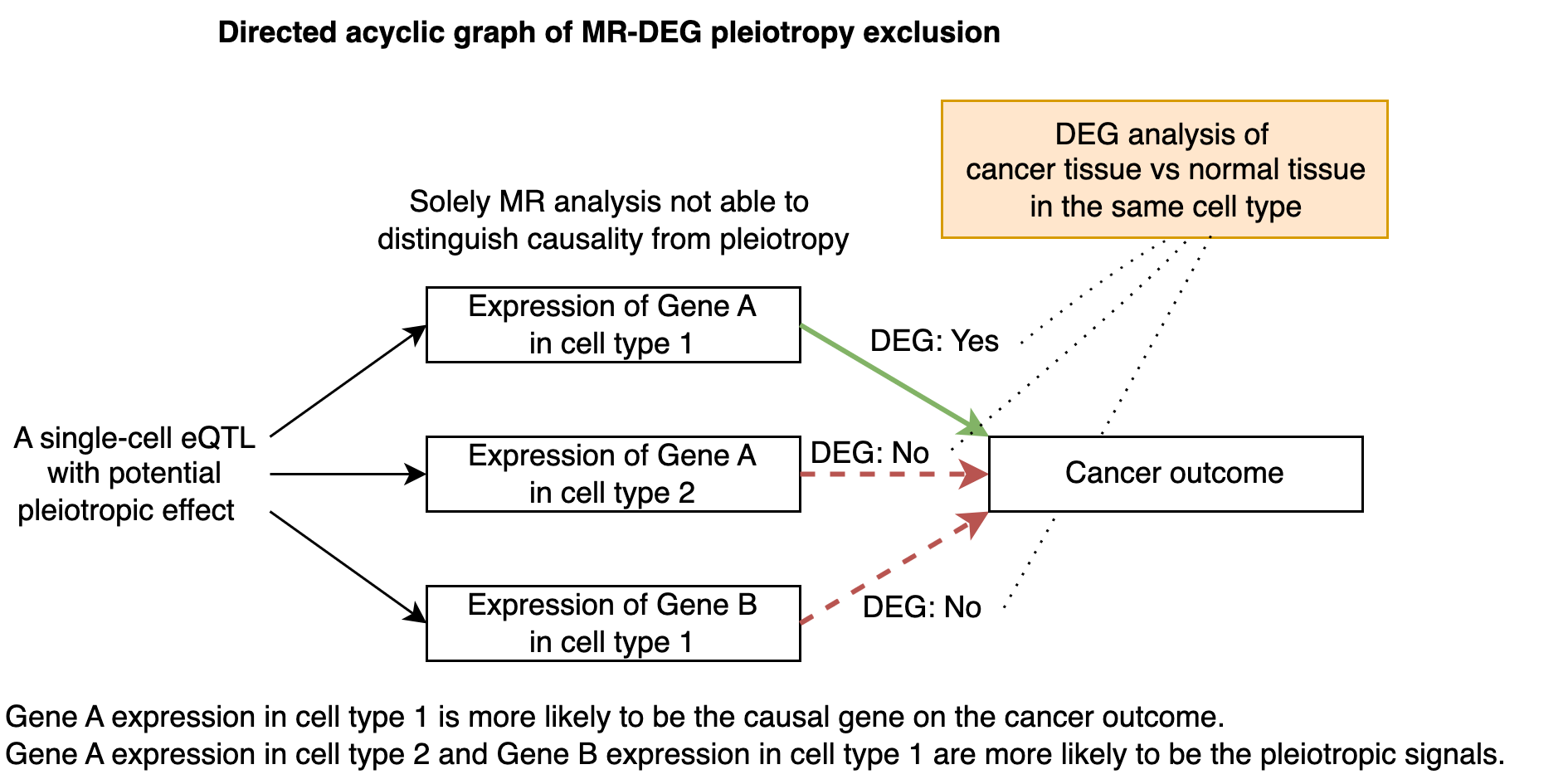


**Figure S3**. Directed acyclic graph of MR-DEG pleiotropy exclusion


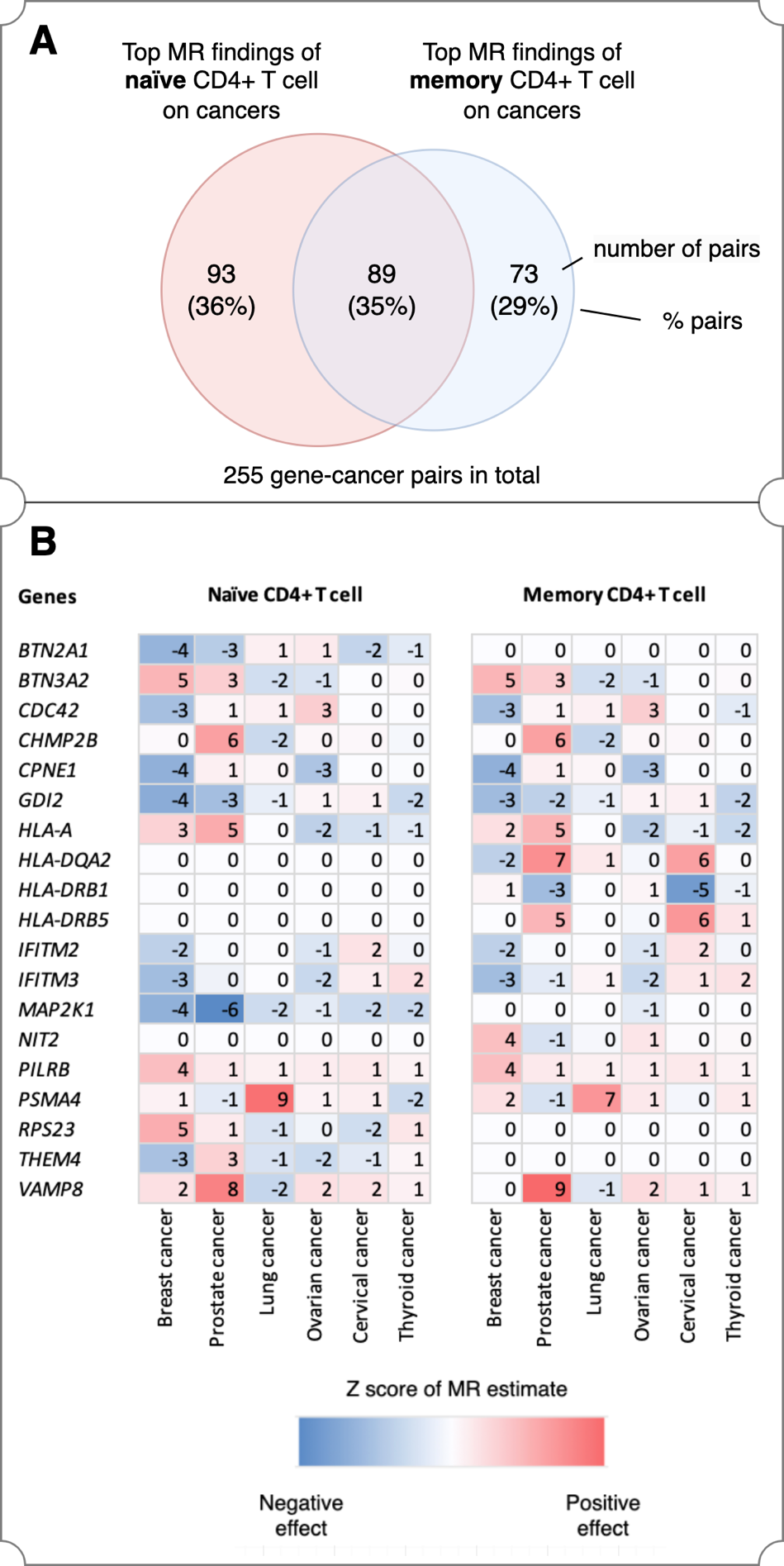


**Figure S4.** Comparison of MR findings in naïve vs memory T cell. (A) Venn diagram of top gene-cancer pairs with MR and colocalization evidence estimated by naïve or memory CD4+ T cell eQTLs. (B) heatmap show the colocalizing diseases and the Z-scores of the MR estimates between gene expressions and cancers in naïve vs memory CD4+ T cells. Red, positive gene-cancer association; blue, negative gene-cancer association.


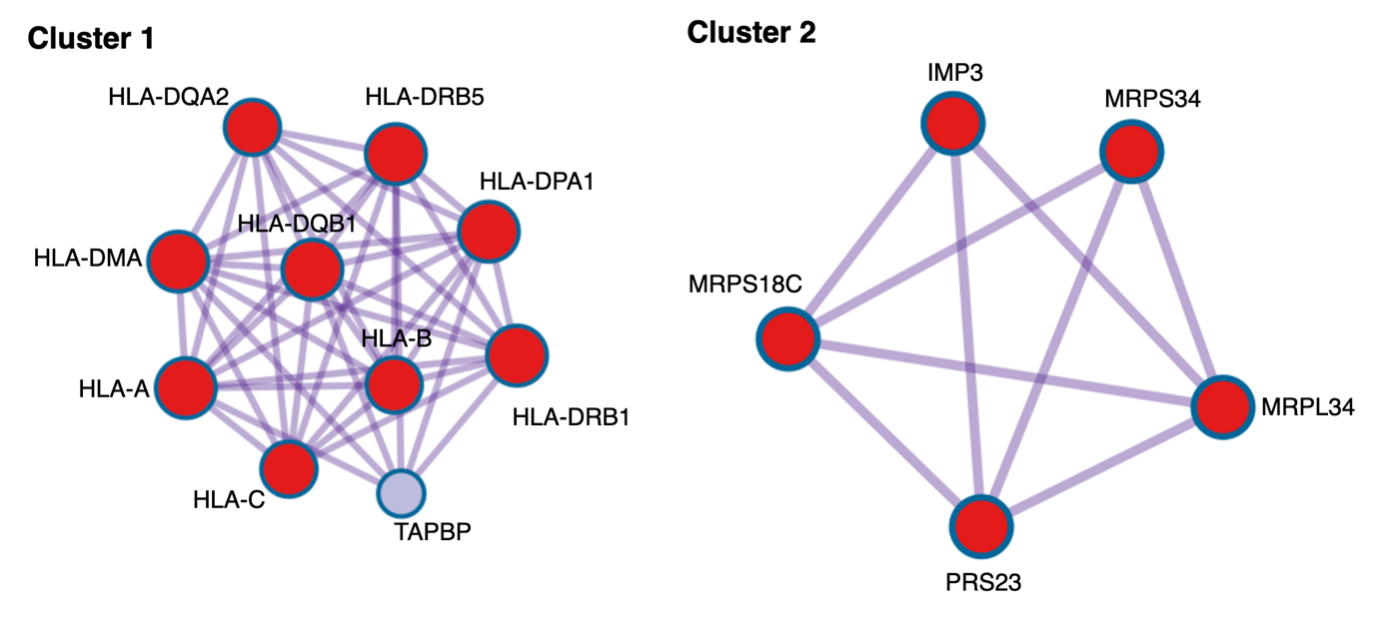


**Figure S5**. Two clusters of genes identified by MCODE.


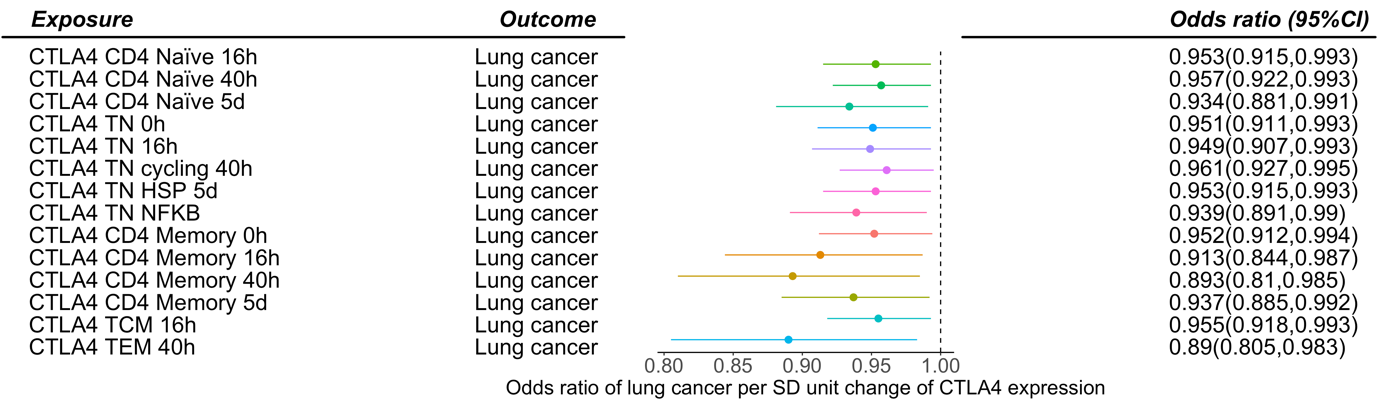


**Figure S6**. Forest plot of effects of *CTLA4* expressions in multiple CD4+ T cells on cancers.
